# Inequalities in provision of hip and knee replacement surgery for osteoarthritis by age, sex, and social deprivation in England between 2007–2017: A population-based cohort study of the National Joint Registry

**DOI:** 10.1371/journal.pmed.1004210

**Published:** 2023-04-27

**Authors:** Erik Lenguerrand, Yoav Ben-Shlomo, Amar Rangan, Andrew Beswick, Michael R. Whitehouse, Kevin Deere, Adrian Sayers, Ashley W. Blom, Andrew Judge

**Affiliations:** 1 Musculoskeletal Research Unit, Translational Health Sciences, Bristol Medical School, University of Bristol, Southmead Hospital, Bristol, United Kingdom; 2 Population Health Sciences, Bristol Medical School, University of Bristol, Bristol, United Kingdom; 3 Department of Health Sciences, University of York, Heslington, York, United Kingdom; 4 National Joint Registry, London, United Kingdom; 5 National Institute for Health Research Biomedical Research Centre at University Hospitals Bristol and Weston NHS Foundation Trust and the University of Bristol, Bristol, United Kingdom; 6 Nuffield Department of Orthopaedics, Rheumatology and Musculoskeletal Sciences, Nuffield Orthopaedic Centre, University of Oxford, Oxford, United Kingdom; Harvard Medical School, UNITED STATES

## Abstract

**Background:**

While the United Kingdom National Health Service aimed to reduce social inequalities in the provision of joint replacement, it is unclear whether these gaps have reduced. We describe secular trends in the provision of primary hip and knee replacement surgery between social deprivation groups.

**Methods and findings:**

We used the National Joint Registry to identify all hip and knee replacements performed for osteoarthritis from 2007 to 2017 in England. The Index of Multiple Deprivation (IMD) 2015 was used to identify the relative level of deprivation of the patient living area. Multilevel negative binomial regression models were used to model the differences in rates of joint replacement. Choropleth maps of hip and knee replacement provision were produced to identify the geographical variation in provision by Clinical Commissioning Groups (CCGs).

A total of 675,342 primary hip and 834,146 primary knee replacements were studied. The mean age was 70 years old (standard deviation: 9) with 60% and 56% of women undergoing hip and knee replacements, respectively. The overall rate of hip replacement increased from 27 to 36 per 10,000 person-years and knee replacement from 33 to 46. Inequalities of provision between the most (reference) and least affluent areas have remained constant for both joints (hip: rate ratio (RR) = 0.58, 95% confidence interval [0.56, 0.60] in 2007, RR = 0.59 [0.58, 0.61] in 2017; knee: RR = 0.82 [0.80, 0.85] in 2007, RR = 0.81 [0.80, 0.83] in 2017). For hip replacement, CCGs with the highest concentration of deprived areas had lower overall provision rates, and CCGs with very few deprived areas had higher provision rates. There was no clear pattern of provision inequalities between CCGs and deprivation concentration for knee replacement.

Study limitations include the lack of publicly available information to explore these inequalities beyond age, sex, and geographical area. Information on clinical need for surgery or patient willingness to access care were unavailable.

**Conclusions:**

In this study, we found that there were inequalities, which remained constant over time, especially in the provision of hip replacement, by degree of social deprivation. Providers of healthcare need to take action to reduce this unwarranted variation in provision of surgery.

## Author summary

**Why was this study done?:**

- Joint replacements are among the more frequent elective surgeries performed in developed settings.
- In England, inequalities in provision of joint replacement surgery were reported more than a decade ago, followed by a national effort to reduce these inequalities.
- In a context of increasingly strained National Health Service funding and hospital budgets, alongside prolonged efforts to increase surgical capacity, it is unclear what impact these have had on inequalities in provision of joint replacement for osteoarthritis.

**What did the researchers do and find?:**

- We conducted an analysis of the National Joint Registry using all primary hip and knee replacements for osteoarthritis performed from 2007 to 2017 in England, merged with the Office for National Statistics official population statistics, to investigate inequalities in provision of surgery according to deprivation by patient area of residence, age, sex, and trends in these inequalities over time.
- Using 675,342 primary hip and 834,146 primary knee replacements, we found that inequalities in provision of surgery between the most (reference) and least affluent areas have remained large (hip: rate ratio (RR) = 0.58, 95% confidence interval [0.56, 0.60] in 2007, RR = 0.59 [0.58, 0.61] in 2017; knee: RR = 0.82 [0.80, 0.85] in 2007, RR = 0.81 [0.80, 0.83] in 2017), despite the use of outsourcing of surgery to the private sector.
- For hip replacement, Clinical Commissioning Groups (CCGs) with the highest concentration of deprived areas had lower overall provision of surgery, and CCGs with very few deprived areas had higher surgical provision.
- There has been an overall improvement in the absolute numbers of hip and knee replacement operation being performed; there is now less geographical variation in rates of surgery. For publicly funded surgery, no socioeconomic inequalities were observed for knee replacements, with a smaller inequality observed for hip replacements, whereas for privately funded surgery, strong inequalities were observed for both joints.

**What do these findings mean?:**

- This study found that socioeconomic inequalities in provision of joint replacement surgery are still prevailing.
- Care providers should take action to address unwarranted variation in the provision of joint replacement surgery.

## Introduction

Primary hip and knee replacements are common elective orthopaedic surgical procedures for the treatment of hip and knee pain due to end stage osteoarthritis [1]. These procedures are highly effective in reducing pain and functional limitations for the vast majority of patients [2–4] and are cost effective [5,6]. Over 200,000 hip and knee replacements are performed each year in the United Kingdom [7]. The lifetime risk in the UK is estimated to be 10.8% for women and 8.1% for men undergoing knee replacement and 11.6% women and 7.1% men for hip replacement [8]. These numbers are projected to increase [9] due to an ageing and increasingly obese population, thereby placing an increasing public health burden on the National Health Service (NHS) in respect of funding and capacity.

Fairness in access to healthcare was one of the founding principles of the UK NHS at its inception in 1948 and remains so today. Commissioners of healthcare, who have responsibility for health services, need to be concerned about the quality of healthcare that they commission, with a focus on reducing unwarranted variations in quality and access [10,11]. There are well-known inequalities and variation in the provision of common surgical procedures including hip and knee replacement [12,13]. In recent years, and before the Coronavirus Disease 2019 (COVID-19) pandemic, treatment capacity in the NHS has not grown fast enough to keep up with patient need, including hip and knee replacements [14]. In February 2010, the report “Fair Society Healthy Lives” focused on evidence-based strategies for reducing health inequalities in England [15]. The past decade has coincided with austerity and increasingly strained NHS funding and hospital budgets, where the health gap has now grown between wealthy and deprived areas [16]. It has previously been shown that older patients, females, and those living in the most deprived areas have the greatest clinical need for both hip and knee replacement surgery [17]. In a study using hospital admission data from 2002, inequity in access to care was observed, with these patient groups least likely to receive access to surgery relative to their clinical need [18].

The current climate is one of increasingly strained NHS funding and hospital budgets. NHS funding growth is now slower than historical trends [19]. Independently of the COVID-19 pandemic, there was an average daily 10,015 occupied beds open overnight for trauma and orthopaedics in England between April and June 2010, dropping to 8,770 between October and December 2016 [20]. Despite there being fewer beds in the NHS, more publicly funded joint replacements are being done due to outsourcing of NHS patients to the independent sector [21,22]. From 2007 to 2017, the total numbers of hip replacements increased from 60,898 to 95,909 and knee replacements from 67,028 to 106,574 [7]. It is unclear in the context of such changes, what impact this has had on inequalities in provision and access to joint replacement in the public and private sectors.

Using data on patients in England from the National Joint Registry, the aim of this study is to compare the change in provision of primary hip and knee replacement surgery for osteoarthritis by social deprivation, age, and sex across England between 2007 and 2017.

## Methods

We conducted a retrospective analysis of prospectively collected anonymised data on patients in England from the National Joint Registry. We used NJR data from 1 January 2007 to 31 December 2017 to identify primary hip and knee procedures performed in England [7]. It is mandatory for surgeons and their hospital to register all hip and knee replacement activity in the NJR whether the procedures are funded by the NHS or independently. The NJR contains anonymised patient data on age, sex, and year of procedure. Information on the residential area of the patient, as defined by the 2011 census Lower Layer Super Output Areas (LSOAs), are also available. Data and related programming files are available from the authors with due to permission from the NJR scientific committee (https://www.njrcentre.org.uk/research/research-requests/).

We used official statistics from the Office for National Statistics (ONS) to identify midyear estimates of the age–sex-specific usual resident population of each 2011 LSOA in England. Information on the level of deprivation was obtained using the Index of Multiple Deprivation (IMD). Each LSOA is assigned a deprivation score and ranked accordingly. We used the 2015 IMD version and derived IMD quintiles, with quintile 1 (IMD1) associated with the most deprived LSOAs and quintile 5 (IMD5) associated with the least deprived LSOAs.

Participants eligible for inclusion in the study were all patients aged 50 years or more, operated on and living at the time of surgery in England, who had received a primary total hip, total or unicompartmental knee replacement between 2007 and 2017 for an indication of osteoarthritis only. This included patients treated in NHS hospitals, independent hospitals, and independent sector treatment centres. Patients living or operated on outside England were excluded, as were those operated on for an indication other than or in addition to osteoarthritis, and/or who had received a revision joint replacement. Finally, patients with no residential area information were excluded.

The primary outcome was the rate of provision of primary hip or knee replacement. Yearly rates of provision were derived by aggregating the NJR patient-level data by year of procedure, age, sex, and LSOA group to obtain the count of hip and knee replacements in each of these strata (numerator) and using the aggregated ONS count of population living in each of these strata (denominator). The rates are reported per 10,000 persons.

The IMD 2015 was used to identify the relative level of deprivation of the patient living area, modelled in quintiles (IMD = 1 (most deprived) to IMD = 5 (least deprived)). Other variables included age group (50 to 59, 60 to 69, 70 to 79, 80+), sex (male, female), and the year of the primary joint replacement (2007 to 2017).

The planning and commissioning of healthcare services for each LSOA is defined at the level of Clinical Commissioning Groups (CCGs), with LSOAs nested within CCG. We used data provided by the ONS to identify the 2011 LSOAs nested in each CCG area [23].

Analyses were stratified according to whether or not patients had received public versus privately funded procedures for their joint replacement, to see if this explained any observed trends in inequalities by social deprivation group over time. Operations performed in the independent sector but funded by the NHS were considered as public funded procedures.

### Statistical analysis

The rates of joint replacement in each IMD group and year of joint replacement, standardised by age and sex, were first produced. Multilevel negative binomial regression models were used to compare these rates, i.e., to produce rate ratios (RRs). These models were chosen to account for overdispersion. We included an offset term to account for the population at risk in each compared group, i.e., the denominators of the modelled rates. Areas of residence (LSOAs) were modelled as random effects. The models were adjusted for deprivation groups (IMD quintiles, measured at the LSOA level from patient postcode), procedure years, patient age, and sex. They were also adjusted for an interaction between year of joint replacement and IMD group to perform within and between comparisons, i.e., investigate the differences in joint provision between IMD groups in a particular year, or the difference in joint provision over time in a particular IMD group, or the difference in provision change over time between IMD groups.

The rates of joint replacement were then produced for each year of joint replacement by age or sex groups, standardised by IMD and sex or age groups. Multilevel negative binomial regression models were also used to compare the rates by age or sex strata.

Hip and knee replacement patients were analysed separately. The results of the univariable and multivariable models are presented in Tables A-C in S1 Appendix.

Geographical variation in hip and knee provision were then considered by CCG area, using the directly standardised age–sex rate of each CCG for 2007, 2012, and 2017 (beginning, middle, and end of study period). The CCG boundaries, as at 1 April 2017, were used to derive choropleth maps of hip and knee replacement provision (obtained from the ONS) [24]. To investigate how deprivation impacted variation in provision rates across CCG areas, we classified the CCGs according to the concentration of LSOA(s) with the highest concentration of deprived areas (percentage of LSOAs with IMD rank in Q1): less than 5% (i.e., 0% to 5% of the LSOAs within a specific CCG have an IMD rank = Q1), 5% to 15%, 15% to 30%, and ≥30%. These thresholds correspond to the quartiles of LSOAs deprivation concentration by CCG. We reported the rates using caterpillar plots.

Analyses were conducted using Stata version 15.1 statistical software (StataCorp, College Station, Texas). Geographical Information System maps were produced with ArcMap 10.6. We followed the STROBE (Strengthening the Reporting of Observational Studies in Epidemiology) guideline to report our study S1 STROBE Checklist [25].

### Ethics approval and consent to participate

With support under Section 251 of the NHS Act 2006, the Ethics and Confidentiality Committee (ECC) (now the Health Research Authority Confidentiality Advisory Group) allows the NJR to collect patient data where consent is indicated as “Not Recorded.”

Before Personal Data and Sensitive Personal Data are recorded, express written patient consent is provided. The NJR records patient consent as either “Yes,” “No,” or “Not Recorded.”

## Results

Details of the study population are provided in **Figures A1 and A2** in S1 Appendix and **Table 1**. A total of 675,342 primary hip and 834,146 primary knee replacements for OA were performed in England between 2007 and 2017 among patients aged 50 years and over. Their mean age was 70 years old (standard deviation: 9) with 60% and 56% of women undergoing hip and knee replacements, respectively. The overall provision of hip replacement increased from 27/10,000 persons in 2007 to 36/10,000 in 2017 and from 33/10,000 persons to 46/10,000 for knee replacement. Rates of both hip and knee replacement were highest for patients living in the least deprived areas, females, and those aged 70 to 80 years old.

**Table 1:** Sample description by joint replacement*.

	Overall				2007				2012				2017			
	Cases	N	Rate	95%CI	Cases	N	Rate	95%CI	Cases	N	Rate	95%CI	Cases	N	Rate	95%CI
**HIP**																
First IMD quintile-most deprived	74,460	33,063,152	22.5	[22.4, 22.7]	5,320	2,841,376	18.7	[18.2, 19.2]	6,845	2,980,592	23.0	[22.4, 23.5]	7,759	3,245,278	23.9	[23.4, 24.4]
Second IMD quintile	107,374	37,536,979	28.6	[28.4, 28.8]	7,863	3,162,909	24.9	[24.3, 25.4]	9,774	3,390,586	28.8	[28.3, 29.4]	11,315	3,721,442	30.4	[29.8, 31.0]
Third IMD quintile	149,824	43,318,183	34.6	[34.4, 34.8]	10,548	3,625,508	29.1	[28.5, 29.6]	13,572	3,919,715	34.6	[34.0, 35.2]	16,231	4,296,269	37.8	[37.2, 38.4]
Fourth IMD quintile	167,980	45,630,603	36.8	[36.6, 37.0]	11,638	3,797,336	30.6	[30.1, 31.2]	15,087	4,135,745	36.5	[35.9, 37.1]	18,467	4,531,015	40.8	[40.2, 41.3]
Fifth IMD quintile-least deprived	175,704	46,482,232	37.8	[37.6, 38.0]	12,027	3,851,272	31.2	[30.7, 31.8]	15,719	4,219,959	37.2	[36.7, 37.8]	19,620	4,605,666	42.6	[42.0, 43.2]
Female	405,314	108,992,020	37.2	[37.1, 37.3]	28,652	9,229,700	31.0	[30.7, 31.4]	36,622	9,858,987	37.1	[36.8, 37.5]	43,975	10,719,937	41.0	[40.6, 41.4]
Male	270,028	97,039,129	27.8	[27.7, 27.9]	18,744	8,048,701	23.3	[23.0, 23.6]	24,375	8,787,610	27.7	[27.4, 28.1]	29,417	9,679,733	30.4	[30.0, 30.7]
<60	100,500	73,346,897	13.7	[13.6, 13.8]	7,411	6,229,272	11.9	[11.6, 12.2]	8,791	6,577,190	13.4	[13.1, 13.6]	11,020	7,386,230	14.9	[14.6, 15.2]
60–70	220,519	62,587,940	35.2	[35.1, 35.4]	15,893	5,154,335	30.8	[30.4, 31.3]	20,229	5,804,951	34.8	[34.4, 35.3]	22,618	5,873,567	38.5	[38.0, 39.0]
70–80	248,881	42,600,041	58.4	[58.2, 58.7]	17,431	3,600,935	48.4	[47.7, 49.1]	22,182	3,756,525	59.0	[58.3, 59.8]	27,680	4,417,956	62.7	[61.9, 63.4]
80+	105,442	27,496,271	38.3	[38.1, 38.6]	6,661	2,293,859	29.0	[28.3, 29.7]	9,795	2,507,931	39.1	[38.3, 39.8]	12,074	2,721,917	44.4	[43.6, 45.1]
**KNEE**																
First IMD quintile-most deprived	115,019	33,063,168	34.8	[34.6, 35.0]	8,038	2,841,377	28.3	[27.7, 28.9]	10,463	2,980,591	35.1	[34.4, 35.8]	12,229	3,245,278	37.7	[37.0, 38.3]
Second IMD quintile	145,914	37,536,998	38.9	[38.7, 39.1]	10,178	3,162,917	32.2	[31.6, 32.8]	13,091	3,390,589	38.6	[37.9, 39.3]	15,923	3,721,444	42.8	[42.1, 43.5]
Third IMD quintile	181,515	43,318,199	41.9	[41.7, 42.1]	12,526	3,625,510	34.5	[33.9, 35.2]	16,406	3,919,713	41.9	[41.2, 42.5]	19,828	4,296,271	46.2	[45.5, 46.8]
Fourth IMD quintile	195,457	45,630,621	42.8	[42.6, 43.0]	13,262	3,797,344	34.9	[34.3, 35.5]	17,408	4,135,745	42.1	[41.5, 42.7]	22,176	4,531,014	48.9	[48.3, 49.6]
Fifth IMD quintile-least deprived	196,241	46,482,244	42.2	[42.0, 42.4]	13,031	3,851,282	33.8	[33.3, 34.4]	17,273	4,219,961	40.9	[40.3, 41.5]	22,766	4,605,665	49.4	[48.8, 50.1]
Female	470,487	108,992,040	43.2	[43.0, 43.3]	32,400	9,229,714	35.1	[34.7, 35.5]	42,176	9,858,988	42.8	[42.4, 43.2]	52,018	10,719,937	48.5	[48.1, 48.9]
Male	363,659	97,039,190	37.5	[37.4, 37.6]	24,635	8,048,716	30.6	[30.2, 31.0]	32,465	8,787,611	36.9	[36.5, 37.3]	40,904	9,679,735	42.3	[41.8, 42.7]
<60	117,825	73,346,897	16.1	[16.0, 16.2]	7,434	6,229,272	11.9	[11.7, 12.2]	10,568	6,577,190	16.1	[15.8, 16.4]	13,872	7,386,230	18.8	[18.5, 19.1]
60–70	291,737	62,587,939	46.6	[46.4, 46.8]	19,541	5,154,335	37.9	[37.4, 38.4]	26,645	5,804,951	45.9	[45.4, 46.5]	31,305	5,873,566	53.3	[52.7, 53.9]
70–80	309,550	42,600,058	72.7	[72.4, 72.9]	22,065	3,600,942	61.3	[60.5, 62.1]	27,145	3,756,523	72.3	[71.4, 73.1]	35,133	4,417,955	79.5	[78.7, 80.4]
80+	115,034	27,496,336	41.8	[41.6, 42.1]	7,995	2,293,881	34.9	[34.1, 35.6]	10,283	2,507,935	41.0	[40.2, 41.8]	12,612	2,721,921	46.3	[45.5, 47.1]

*The statistics are provided for the overall observation period (2007–2017) and for the beginning (2007), middle (2015), and end (2017) of this observation period.

The rates and related 95% confidence intervals (95% CI) are per 10,000 persons.

### Sex

Males received lower rates of hip replacement than females **(Figure B** in S1 Appendix). The relative sex gap has remained constant over time for hip replacements in 2007 (RR = 0.78, 95% confidence interval [0.77, 0.79], *p* < 0.001) and in 2017 (RR = 0.76 [0.75, 0.77], *p* < 0.001) **(Figure C** in S1 Appendix**)**. The lower provision of knee replacement observed for males in 2007 (RR = 0.90 [0.88, 0.91], *p* < 0.001) was still evident in 2017 (RR = 0.88 [0.87, 0.90], *p* < 0.001).

### Age

The provision of hip and knee replacements was higher for those aged 70 to 80 years old (**Figure D** in S1 Appendix) compared to all other age groups **(Figure E** in S1 Appendix**)**.

### IMD deprivation

**Fig 1:**
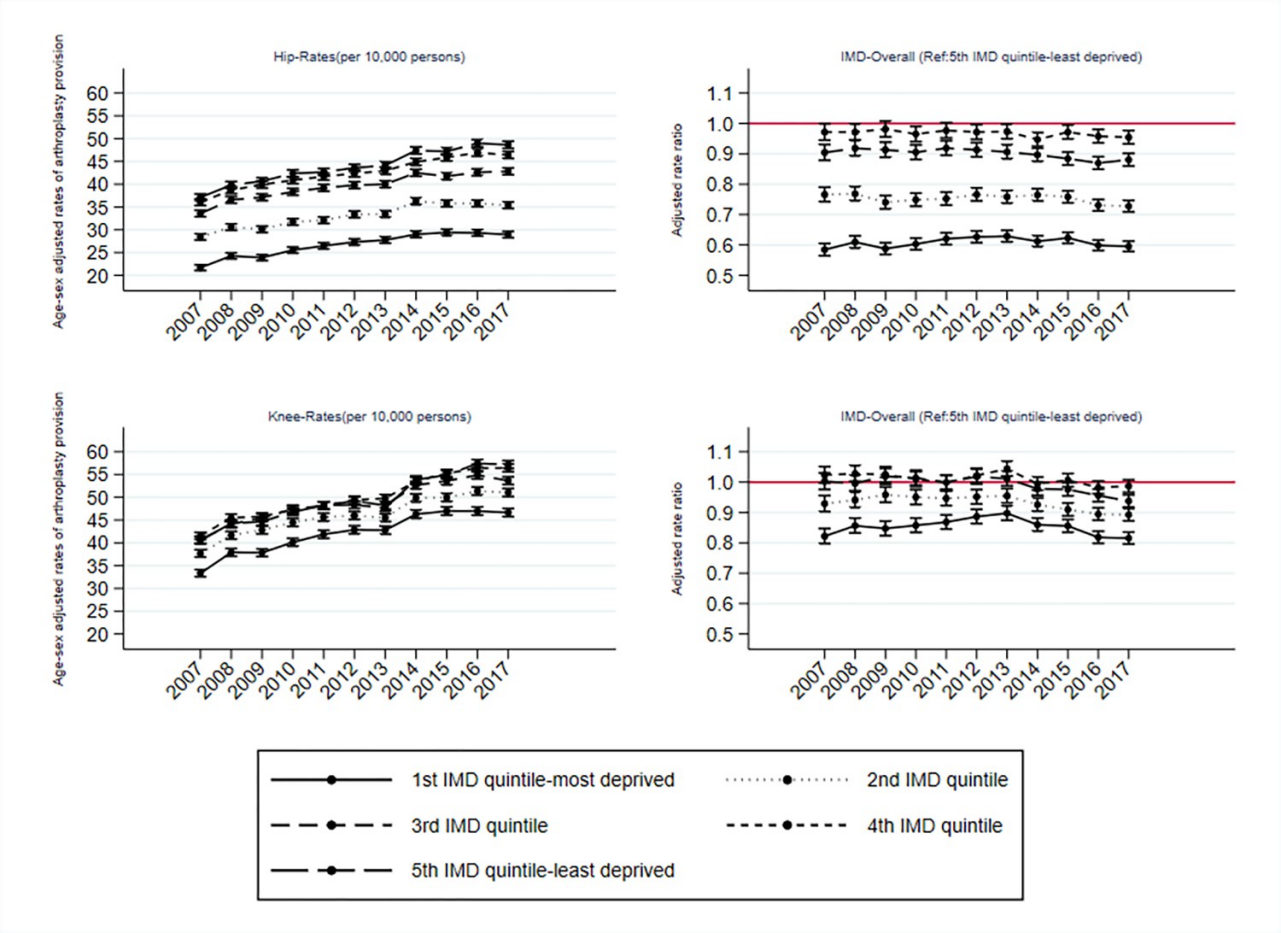
Rates and RRs with 95% confidence intervals of joint replacement provision by area of residence deprivation level adjusted for age, sex, and area of residence (LSOA). IMD, Index of Multiple Deprivation; LSOA, Lower Layer Super Output Area; RR, rate ratio.

**Fig 1** describes the trends in rates of provision between 2007 and 2017 by IMD deprivation group. For hip replacement, the inequalities in access to care have remained with no change over time in the extent of the relative differences between the most and least deprived groups (IMD 1 and 5). The gap between the least deprived group (IMD 5) and IMD 2 (*p* = 0.004) or IMD 3 (*p* = 0.025) widened between 2012 and 2017.

Between 2007 and 2017, the RRs of knee replacement decreased for all IMD groups (IMD 1 to 3 *p* < 0.001, IMD 4 *p* = 0.019) compared to the least deprived group (IMD 5). From 2007 to 2013, the gap had marginally narrowed, but from 2014 onward, it had widened again.

When stratifying these trends by sex **(Figure F** in S1 Appendix**)**, the patterns seen for IMD deprivation were the same for hip and knee replacement. For knee replacement **(Figure G** in S1 Appendix**)**, the inequalities were stronger (*p* < 0.001) and had widened more over time in the male population (*p* < 0.001).

The pattern of inequality by IMD deprivation group for hip replacement was consistent when stratified by type of funding with evidence of disparities observed for both publicly and privately funded procedures **(Figure H** in S1 Appendix**)**. For knee replacement, inequalities were only observed for the privately funded procedures.

The pattern of inequality by IMD deprivation group for hip replacement was consistent when stratified by age groups **(Figure I1** in S1 Appendix**)**, with larger and widening inequalities between the most and least deprived groups observed among older patients (70 years and older) than younger patients (<70 years old) (*p* < 0.001) **(Figure J1** in S1 Appendix**)**. For knee replacement, no pattern of inequalities was observed among those aged 60 years old or younger with the lowest provision in the least deprived group **(Figures I2 and J2** in S1 Appendix**)**. For patients aged 60 to 70 years old, inequalities were seen from 2014 onwards with the lowest provision for those in IMD groups 1 and 2 (most deprived). For those aged 70 years old or over, compared to those in the least deprived groups, people in the 2 most deprived groups (IMD 1 and 2) have had a lower knee replacement provision since 2007 and inequalities started to be seen in the IMD groups 3 and 4 in 2017.

### Geographical variation by Clinical Commissioning Groups (CCGs)

**Fig 2:**
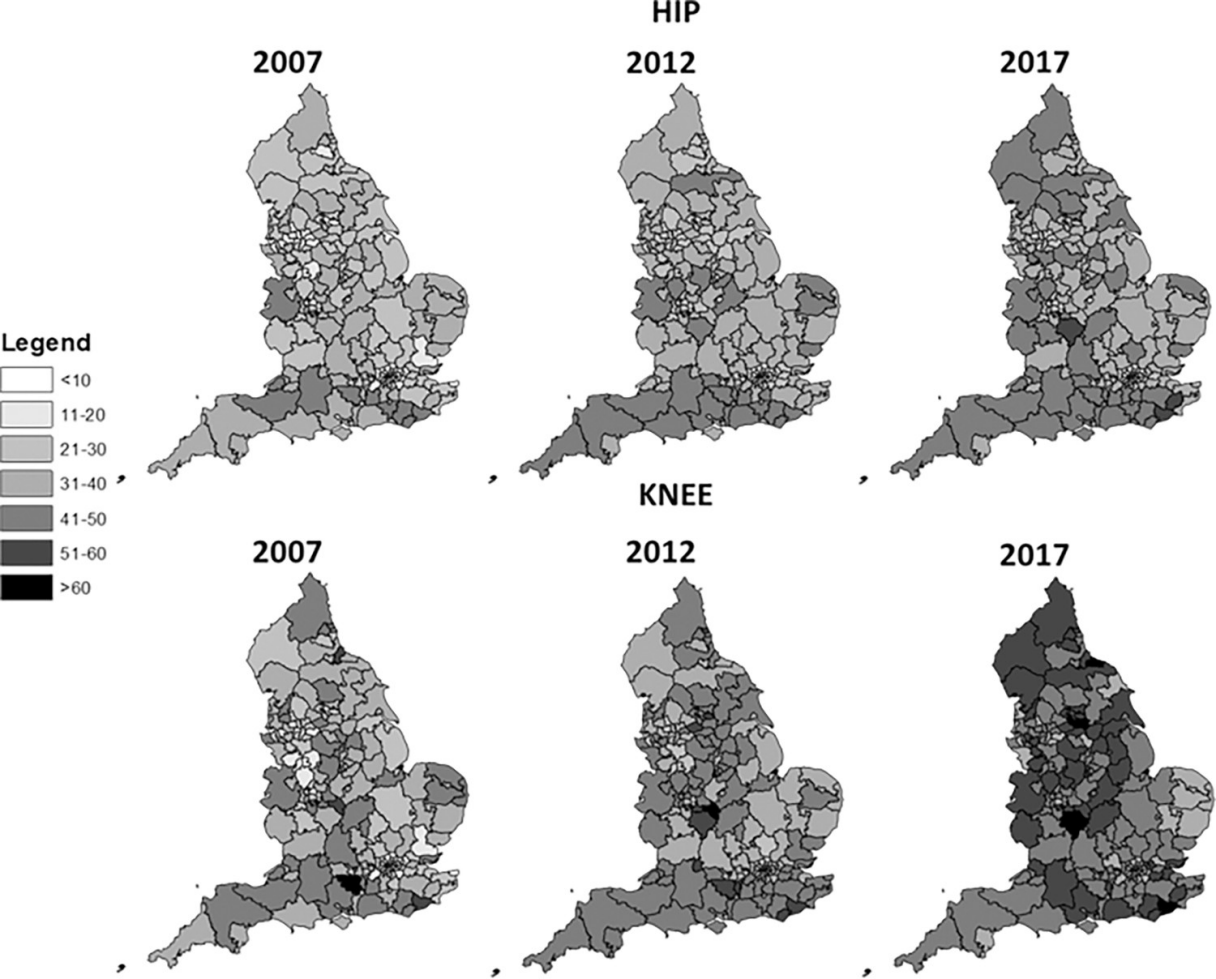
Directly standardised age–sex rates of joint replacement per 10,000 persons within Commissioning Care Groups*.The digital vector boundaries for Clinical Commissioning Groups (CCG), in England, as at 1 April 2017, are available under the Open Government Licence v3.0 (Contains public sector information licensed under the Open Government Licence v3.0; see https://www.nationalarchives.gov.uk/doc/open-government-licence/version/3/). *Clinical Commissioning Groups (April 2017) Boundaries (Version 4). Available from: https://www.data.gov.uk/dataset/2f9234c2-2798-4fbf-b030-05119b42ccb6/clinical-commissioning-groups-april-2017-boundaries-version-4.

The age- and sex-adjusted rates of hip and knee provision for the 207 CCGs for 2007, 2012, and 2017 are presented in **Fig 2**. The provision of hip and knee replacement has increased unequally over time across commissioning group areas in England. In 2007, the overall variation in rates of provision of hip replacement was 16-fold ranging from 2.9/10,000 to 46.5/10,000 across the CCG areas, but by 2017, the amount of geographical variation had decreased to be around 4-fold from 11.7/10,000 to 51.4/10,000. For knee replacement, variation across CCGs in provision ranged from 4.9/10,000 to 61.2/10,000 in 2007 and 20.0/10,000 to 66.4/10,000 in 2017.

**Fig 3:**
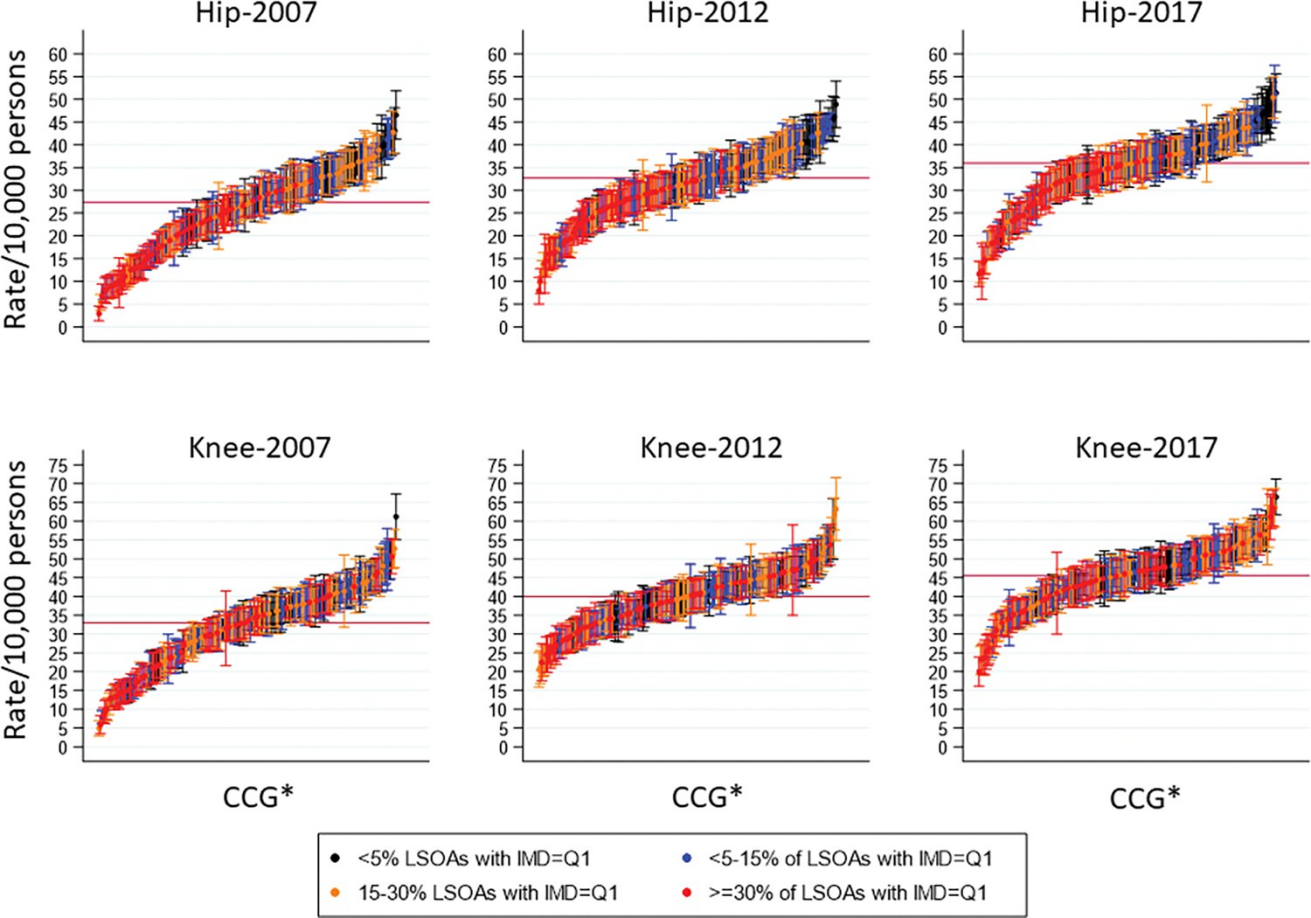
Rates with 95% confidence intervals of joint replacement provision per 10,000 persons for each CCG*, by concentration of deprived area of residence (LSOA with IMD = 1, most deprived). CCG, Clinical Commissioning Group; IMD, Index of Multiple Deprivation; LSOA, Lower Layer Super Output Area.

For hip replacement, CCGs with the highest concentration of deprived LSOAs had provision rates below the annual national rate **(Fig 3)**. This was true for each reported time-period. On the contrary, CCGs with very few deprived areas were more likely to have a higher provision rate above the average national rate, and this is particularly evident in 2017. For knee replacement, there is no clear pattern of provision inequalities between CCGs and their status of deprivation concentration.

## Discussion

Using data from a large national linked dataset of patients receiving primary hip and knee replacement surgery, we observed evidence of inequalities by social deprivation. The relative inequality gap by social deprivation group between the most versus least affluent areas have remained constant over time for both joints. When stratifying the effect of social deprivation by sex, the patterns seen for IMD deprivation were the same for hip and knee replacement. For knee replacement, the social deprivation inequalities were only observed for the privately funded procedures, whereas for hip replacement, evidence of disparities was observed for both publicly and privately funded procedures. The pattern of inequality by IMD deprivation group for hip replacement was consistent when stratified by age groups, i.e., was larger among older patients (70 years and older) than younger patients (<70 years old). For knee replacement, no pattern of inequalities was observed among those aged 60 years old or younger.

Strengths of this study include the use of a large mandatory national dataset, capturing approximately 95% of all such procedures [7]. Data completion and accuracy are excellent for procedures recorded within the NJR [26]. The NJR captures operations for patients treated in NHS and independent hospitals along with independent sector treatment centres, and for this study, the IMD 2015 deprivation index was linked to all patients in the NJR based on the LSOA a patient lived in. Previous studies using data from Hospital Episode Statistics could only explore inequalities for NHS patients [18]. Denominator data used to calculate rates was obtained from the ONS, with these population counts obtained for each year. A strength of the study was having a decade of data to monitor trends in inequalities over a period of time that included publication of the Marmot review [15] and national focus on health inequalities, alongside austerity and increasingly strained NHS funding and hospital budgets. Limitations of the study are that we were only able to look at inequalities according to age, sex, area deprivation at the LSOA level, and geographical area at the CCG level. The reason being that we were limited by data on population counts only being available by age, sex, year, and geographical area. Hence, we could not describe inequalities by other important domains at the patient level such as ethnicity, body mass index, social class, income, and education. Further limitations were that we could not adjust for measures of clinical need for surgery, nor patient willingness to access care. This study is focussed on inequalities in the provision of joint replacement rather than about the inequalities in clinical need. Finally, this research has focused on the most frequent indication for joint replacement, around 90% of the primary operations are performed for osteoarthritis, and in patients aged 50 years or older in the UK [7,27]; the presented results do not apply to those younger or operated for another indication.

Our findings are consistent with previous research where rates of joint replacement increase with age before falling in the oldest age groups [12,28,29], women receive more operations than men [12,28,29], and more affluent groups receive greater provision [12,28,30,31]. Our findings also highlight the expected social differences in the private sector but also reveal the need for tailored public health intervention to reduce social inequality in the provision of hip replacement in the public sector in the lowest social class operated.

Previous studies have shown that clinical need for both hip and knee replacements increase with age up to age 84, before decreasing in those aged over 85 [17,32–34]. The rates of provision in 2016 and 2017 have remained constant for hip replacement or decreased for knee replacement, especially compared to those aged <60 years old. This is despite an increase in the oldest age groups in the general population [35]. These figures are therefore suggestive of inequity for the oldest age groups noted in previous studies [18,36]. Women have a greater clinical need for hip and knee replacement [17], and this reflects patterns of provision we observe.

People living in the most deprived areas have greatest clinical need for surgery [17]. The NHS has opted to deliver part of its orthopaedic activities, funded by public resources, within the private sector. This approach has not modified the inequalities previously reported. We have identified striking, but expected, socioeconomic differences in the procedures privately funded and also in the public sector for hip procedures. Differences between the findings for hip and knee joints may be partly explained by the outcomes of hip replacement surgery being more successful, where 10% of patients are not satisfied after hip replacement compared to up to 20% for knee [37,38], differences in patient willingness of those in more deprived areas to want hip versus knee surgery, and the impact of the symptoms and disabling nature of the disease in the different joints meaning patients may be more or less likely to seek help. Lessons could be learnt from the provision of knee replacements funded by the public sector where no socioeconomic inequalities were identified. Long-term underprovision of hip replacement could explain these inequalities, in which clinical need is known to be higher in the most deprived groups, which, in turn, were least able to privately fund these procedures. On the other hand, this could demonstrate evidence of the Inverse Care Law [39], where the availability of medical care varies inversely with the need of the population served (which would apply just as well to both hip and knee joints). This is consistent with the recent report on health equity 10 years on from the Marmot review of growing inequalities in health according to deprivation and region [16]. Previous studies have suggested the observed inequities by social deprivation may be partly explained by people from more deprived areas being less willing to seek help and access for joint replacement surgery [40], as they are less positive about the benefits and outcomes of surgery, with the decision influenced by friends and family and experiences of those who have had surgery rather than opinions of health professionals. A Canadian study showed that after adjusting for patient willingness, inequity according to patient education was no longer observed [36]. This is unlikely to explain all the reported social inequalities and the NHS needs to organise the delivery of joint replacement to guarantee equality between patients.

Lower social deprivation groups have poorer health status than those in higher social classes [16], but it is assumed that those undergoing joint replacement are more likely to be fitter than their peers in the same social class. This health selection effect may explain why no inequality was observed between patients aged <60 years old. Beyond this age, and despite better health status than their peers from the same social class making them eligible for surgery, lower social class patients had systematically the lowest joint replacement provision. These were already evident in the previous 2 decades [17,18], and these inequalities are still strong today, especially for the lowest social classes in the oldest age groups. Further work will be required to understand the impact of COVID-19 on these inequalities, and it is likely that they have either persisted or increased.

## Conclusions

As common elective procedures, large inequalities by social deprivation group exist in the delivery of hip and knee replacement surgery [13,41–45]. Although the absolute number of these procedures has increased over time, and geographical variation in rates of surgery across commissioning groups areas has declined over time, there is still evidence of socioeconomic inequalities. For publicly funded surgery, no socioeconomic inequalities were observed for knee replacements, with a smaller inequality observed for hip replacements, whereas for privately funded surgery, strong evidence of inequalities were observed for both hip and knee replacements. The relative gap between those in the most affluent and poorest areas has been constant over time. This is inverse to health need where we know those living in the poorest areas have the greatest clinical need for hip and knee replacement surgery. For hip replacement, variation in rates of provision by CCG area was ecologically related to the concentration of deprived areas within a CCG, where those with the highest concentration of deprived areas had the lowest rates of provision. This was not observed for knee replacement, suggesting that inequalities in care provision can be improved for hip replacement. The findings will be informative to commissioners of healthcare to identify how joint replacement provision should be provided in the NHS to best address these disparities.

## Supporting information

S1 STROBE Checklist STROBE statement. (DOCX)

S1 Appendix Figure A1. Patient flow diagram for hip replacement. Figure A2. Patient flow diagram for knee replacement. Figure B. Rates and 95% confidence intervals of joint replacement provision by sex and year of procedure. Figure C. Rates ratio and 95% confidence intervals of joint replacement for males (reference: females) adjusted for age, deprivation, and area of residence (Lower Layer Super Output Area). Figure D. Rates and 95% confidence intervals of joint replacement provision by age and year of procedure. Figure E. Rates ratio and 95% confidence intervals of joint replacement between age groups (reference: <60 years old) adjusted for sex, deprivation, and area of residence (Lower Layer Super Output Area). Figure F. Rates with 95% confidence intervals of joint replacement provision by area of residence deprivation level and year of procedure by sex. Figure G. Rates ratio and 95% confidence intervals of joint replacement between level of area of residence deprivation level (reference: IMD = 5) stratified by sex and adjusted for age and area of residence (Lower Layer Super Output Area). Figure H. Rates ratio and 95% confidence intervals of joint replacement between level of area of residence deprivation level (reference: IMD = 5) stratified by type of healthcare provider and adjusted for age, sex, and area of residence (Lower Layer Super Output Area). Figure I1. Rates with 95% confidence intervals of hip replacement provision by area of residence deprivation level and year of procedure stratified by age groups. Figure I2. Rates with 95% confidence intervals of knee replacement provision by area of residence deprivation level and year of procedure stratified by age groups. Figure J1. Rates ratio and 95% confidence intervals of hip replacement between level of area of residence deprivation level (reference: IMD = 5) stratified by age groups and adjusted for sex and area of residence (Lower Layer Super Output Area). Figure J2. Rates ratio and 95% confidence intervals of knee replacement between level of area of residence deprivation level (reference: IMD = 5) stratified by age groups and adjusted for sex and area of residence (Lower Layer Super Output Area).
(DOCX)

## Abbreviations

CCG: Clinical Commissioning Group
COVID-19: Coronavirus Disease 2019
IMD: Index of Multiple Deprivation
LSOA: Lower Layer Super Output Area
NHS: National Health Service
ONS: Office for National Statistics
RR: rate ratio
