# Supplementary material for: Inequalities in provision of hip and knee replacement surgery for osteoarthritis by age, sex, and social deprivation in England between 2007–2017: A population-based cohort study of the National Joint Registry
Source: PLoS Med. 2023 Apr 27;20(4):e1004210. doi: 10.1371/journal.pmed.1004210 (PMC10138460; doi:10.1371/journal.pmed.1004210)

**Table A.** Adjusted rates of joint replacement by level of Index of Multiple Deprivation (IMD) in the overall population and patient subgroups in 2007, 2012 and 2017

|  |  | **Hip** |  |  |  |  |  |  | **Knee** |  |  |  |  |  |  |
| --- | --- | --- | --- | --- | --- | --- | --- | --- | --- | --- | --- | --- | --- | --- | --- |
|  |  | **2007** |  | **2012** |  | **2017** |  |  | **2007** |  | **2012** |  | **2017** |  |  |
|  |  | **Rate** | **95% CI** | **Rate** | **95% CI** | **Rate** | **95% CI** | **p-value*** | **Rate** | **95% CI** | **Rate** | **95% CI** | **Rate** | **95% CI** | **p-value*** |
| **Overall** | |  |  |  |  |  |  |  |  |  |  |  |  |  |  |
| Model 1 |  |  |  |  |  |  |  | <0.001 |  |  |  |  |  |  | <0.001 |
|  | IMD 1 | 22.2 | [21.5, 23.0] | 27.4 | [26.5, 28.3] | 28.6 | [27.7, 29.5] |  | 24.4 | [23.7, 25.1] | 30.8 | [30.1, 31.6] | 33.2 | [32.4, 34.0] |  |
|  | IMD 2 | 28.9 | [28.0, 29.8] | 33.7 | [32.7, 34.7] | 35.8 | [34.8, 36.8] |  | 27.3 | [26.6, 28.0] | 33.3 | [32.5, 34.1] | 37.0 | [36.2, 37.9] |  |
|  | IMD 3 | 33.7 | [32.8, 34.7] | 40.4 | [39.4, 41.5] | 44.1 | [42.9, 45.2] |  | 29.2 | [28.5, 30.0] | 35.9 | [35.1, 36.7] | 39.6 | [38.8, 40.5] |  |
|  | IMD 4 | 35.9 | [34.9, 36.9] | 43.1 | [[42.0, 44.2] | 48.1 | [46.9, 49.4] |  | 29.6 | [29.0, 30.3] | 36.1 | [35.3, 36.9] | 42.0 | [41.2, 42.9] |  |
|  | IMD 5 | 36.6 | [35.6, 37.6] | 44.1 | [42.9, 45.2] | 50.4 | [49.2, 51.7] |  | 28.7 | [28.0, 29.4] | 35.2 | [34.4, 36.0] | 42.6 | [41.7, 43.5] |  |
| Model 2 |  |  |  |  |  |  |  | <0.001 |  |  |  |  |  |  | <0.001 |
|  | IMD 1 | 16.9 | [16.3, 17.5] | 21.2 | [20.5, 22.0] | 22.4 | [21.7, 23.2] |  | 17.9 | [17.5, 18.5] | 23.1 | [22.5, 23.7] | 25.1 | [24.5, 25.8] |  |
|  | IMD 2 | 22.1 | [21.4, 22.9] | 25.9 | [25.1, 26.8] | 27.4 | [26.6, 28.3] |  | 20.3 | [19.8, 20.9] | 24.8 | [24.1, 25.4] | 27.4 | [26.8, 28.2] |  |
|  | IMD 3 | 26.1 | [25.3, 27.0] | 30.9 | [30.0, 31.9] | 33.1 | [32.2, 34.2] |  | 21.9 | [21.3, 22.5] | 26.5 | [25.9, 27.2] | 28.9 | [28.2, 29.6] |  |
|  | IMD 4 | 28.0 | [27.1, 28.9] | 32.8 | [31.9, 33.9] | 36.0 | [34.9, 37.1] |  | 22.4 | [21.8, 23.0] | 26.6 | [26.0, 27.3] | 30.4 | [29.7, 31.1] |  |
|  | IMD 5 | 28.8 | [27.9, 29.7] | 33.8 | [32.8, 34.8] | 37.7 | [36.5, 38.8] |  | 21.9 | [21.3, 22.4] | 26.0 | [25.4, 26.7] | 30.8 | [30.1, 31.5] |  |
| Model 3 |  |  |  |  |  |  |  | <0.001 |  |  |  |  |  |  | <0.001 |
|  | IMD 1 | 26.2 | [25.3, 27.1] | 32.3 | [31.3, 33.3] | 33.8 | [32.8, 34.9] |  | 26.1 | [25.4, 26.9] | 33.0 | [32.2, 33.9] | 35.6 | [34.7, 36.4] |  |
|  | IMD 2 | 34.0 | [32.9, 35.1] | 39.8 | [38.6, 40.9] | 42.3 | [41.1, 43.5] |  | 29.2 | [28.5, 30.0] | 35.7 | [34.8, 36.5] | 39.7 | [38.8, 40.6] |  |
|  | IMD 3 | 39.8 | [38.7, 41.0] | 47.8 | [46.5, 49.1] | 52.1 | [50.7, 53.5] |  | 31.3 | [30.6, 32.1] | 38.5 | [37.6, 39.3] | 42.5 | [41.6, 43.4] |  |
|  | IMD 4 | 42.4 | [41.2, 43.6] | 50.9 | [49.5, 52.3] | 56.9 | [55.4, 58.4] |  | 31.8 | [31.0, 32.5] | 38.7 | [37.8, 39.5] | 45.1 | [44,1, 46.0] |  |
|  | IMD 5 | 43.3 | [42.1, 44.5] | 52.1 | [50.7, 53.5] | 59.6 | [58.1, 61.2] |  | 30.7 | [30.0, 31.5] | 37.7 | [36.9, 38.6] | 45.7 | [44.7, 46.6] |  |
| Model 4 |  |  |  |  |  |  |  | <0.001 |  |  |  |  |  |  | <0.001 |
|  | IMD 1 | 19.9 | [19.2, 20.7] | 25.1 | [24.2, 26.0] | 26.6 | [25.7, 27.5] |  | 19.1 | [18.6, 19.7] | 24.6 | [23.9, 25.3] | 26.8 | [26.1, 27.5] |  |
|  | IMD 2 | 26.1 | [25.2, 27.0] | 30.7 | [29.6, 31.7] | 32.5 | [31.4, 33.6] |  | 21.6 | [21.0, 22.2] | 26.4 | [25.7, 27.1] | 29.3 | [28.6, 30.0] |  |
|  | IMD 3 | 30.8 | [29.8, 31.9] | 36.5 | [35.4, 37.7] | 39.3 | [38.1, 40.6] |  | 23.3 | [22.7, 24.0] | 28.2 | [27.5, 28.9] | 30.8 | [30.0, 31.5] |  |
|  | IMD 4 | 33.1 | [32.1, 34.2] | 38.9 | [37.7, 40.1] | 42.6 | [41.3, 43.9] |  | 23.8 | [23.2, 24.4] | 28.3 | [27.6, 29.0] | 32.9 | [31.6, 33.2] |  |
|  | IMD 5 | 34.1 | [33.0, 35.2] | 40.0 | [38.8, 41.3] | 44.7 | [43.3, 46.0] |  | 23.3 | [22.7, 23.9] | 27.7 | [27.1, 28.4] | 32.8 | [32.1, 33.6] |  |
| **Within patients <60y old** | |  |  |  |  |  |  |  |  |  |  |  |  |  |  |
| Model 5 |  |  |  |  |  |  |  | 0.113 |  |  |  |  |  |  | <0.001 |
|  | IMD 1 | 13.8 | [12.7, 15.0] | 16.2 | [15.0, 17.4] | 16.8 | [15.7, 18.1] |  | 13.9 | [13.0, 14.9] | 20.5 | [19.3, 21.8] | 21.8 | [20.6, 23.1] |  |
|  | IMD 2 | 16.2 | [15.0, 17.5] | 18.8 | [17.5, 20.3] | 20.1 | [18.8, 21.6] |  | 14.5 | [13.6, 15.5] | 19.2 | [18.1, 20.4] | 22.0 | [20.8, 23.2] |  |
|  | IMD 3 | 19.0 | [17.7, 20.4] | 20.8 | [19.4, 22.2] | 24.4 | [22.8, 26.0] |  | 13.8 | [12.9, 14.7] | 19.7 | [18.6, 20.9] | 21.5 | [20.3, 22.7] |  |
|  | IMD 4 | 19.3 | [18.0, 20.7] | 22.7 | [21.2, 24.2] | 24.3 | [22.8, 25.9] |  | 13.9 | [13.1, 14.9] | 18.0 | [17.0, 19.1] | 22.5 | [21.3, 23.7] |  |
|  | IMD 5 | 19.0 | [17.7, 20.4] | 20.6 | [19.3, 22.1] | 25.0 | [23.4, 26.6] |  | 12.2 | [11.4, 13.0] | 15.6 | [14.7, 16.6] | 21.0 | [19.9, 22.2] |  |
| Model 6 |  |  |  |  |  |  |  | 0.113 |  |  |  |  |  |  | <0.001 |
|  | IMD 1 | 14.4 | [13.2, 15.6] | 16.8 | [15.5, 18.1] | 17.5 | [16.2, 18.8] |  | 15.9 | [14.9, 17.1] | 23.5 | [22.1, 25.0] | 25.0 | [23.7, 26.4] |  |
|  | IMD 2 | 16.8 | [15.6, 18.2] | 19.6 | [18.2, 21.0] | 20.9 | [19.5, 22.4] |  | 16.6 | [15.5, 17.8] | 22.0 | [20.7, 23.4] | 25.2 | [23.8, 26.6] |  |
|  | IMD 3 | 19.7 | [18.3, 21.1] | 21.5 | [20.1, 23.1] | 25.3 | [23.7, 27.0] |  | 15.8 | [14.8, 16.9] | 22.6 | [21.2, 23.9] | 24.6 | [23.3. 26.0] |  |
|  | IMD 4 | 20.0 | [18.7, 21.5] | 23.5 | [22.0, 25.1] | 25.2 | [23.6, 26.9] |  | 16.0 | [15.0, 17.0] | 20.6 | [19.4, 21.9] | 25.7 | [24.3, 27.1] |  |
|  | IMD 5 | 19.7 | [18.3, 21.1] | 21.4 | [20.0, 22.9] | 25.9 | [24.3, 27.6] |  | 13.9 | [13.0, 14.9] | 17.8 | [16.8, 19.0] | 24.0 | [22.7, 25.4] |  |
| **Within patients 60-70y old** | |  |  |  |  |  |  |  |  |  |  |  |  |  |  |
| Model 5 |  |  |  |  |  |  |  | 0.110 |  |  |  |  |  |  | 0.001 |
|  | IMD 1 | 35.9 | [33.5, 38.5] | 43.4 | [40.7, 46.3] | 46.8 | [44.0, 49.9] |  | 41.6 | [39.7, 44.1] | 53.1 | [50.8, 55.8] | 59.0 | [56.3, 61.9] |  |
|  | IMD 2 | 46.3 | [43.4, 49.3] | 51.4 | [48.4, 54.6] | 55.0 | [51.8, 58.4] |  | 45.8 | [43.5, 48.1] | 55.8 | [53.2, 58.5] | 63.0 | [60.2, 65.9] |  |
|  | IMD 3 | 53.8 | [50.7, 57.1] | 60.8 | [57.4, 64.4] | 65.9 | [62.2, 69.8] |  | 47.3 | [45.1, 49.6] | 57.5 | [54.9, 60.1] | 65.4 | [62.6, 68.4] |  |
|  | IMD 4 | 55.7 | [52.5, 59.1] | 63.3 | [59.8, 67.0] | 71.4 | [67.5, 75.5] |  | 47.1 | [45.0, 49.5] | 57.0 | [54.5, 59.6] | 68.0 | [65.1, 71.0] |  |
|  | IMD 5 | 58.4 | [55.1, 61.9] | 64.9 | [61.3, 68.7] | 75.5 | [71.4, 79.8] |  | 45.1 | [43.0, 47.3] | 54.6 | [52.2, 57.1] | 66.8 | [63.9, 69.8] |  |
| Model 6 |  |  |  |  |  |  |  | 0.098 |  |  |  |  |  |  | 0.001 |
|  | IMD 1 | 42.1 | [39.2, 45.2] | 51.0 | [47.7, 54.5] | 55.0 | [51.5, 58.7] |  | 44.2 | [41.9, 46.6] | 56.1 | [53.4, 58.9] | 62.3 | [59.4, 65.4] |  |
|  | IMD 2 | 54.3 | [50.9, 57.9] | 60.3 | [56.6, 64.2] | 64.5 | [60.7, 68.6] |  | 48.3 | [45.9, 50.8] | 58.9 | [56.2, 61.7] | 66.5 | [63.5, 69.6] |  |
|  | IMD 3 | 63.1 | [59.3, 67.1] | 71.2 | [67.1, 75.6] | 77.2 | [72.8, 81.9] |  | 49.9 | [47.6, 52.4] | 60.7 | [58.0, 63.5] | 69.0 | [66.1, 72.2] |  |
|  | IMD 4 | 65.3 | [61.4, 69.4] | 74.2 | [70.0, 78.7] | 83.6 | [78.9, 88.6] |  | 49.8 | [47.5, 52.2] | 60.1 | [57.5, 62.9] | 71.7 | [68.6, 74.9] |  |
|  | IMD 5 | 68.5 | [64.5, 72.7] | 76.1 | [71.7, 80.7] | 88.4 | [83.4, 93.7] |  | 47.6 | [45.4, 50.0] | 57.6 | [55.1, 60.3] | 70.5 | [67.4, 73.6] |  |
| **Within patients 70-80y old** | |  |  |  |  |  |  |  |  |  |  |  |  |  |  |
| Model 5 |  |  |  |  |  |  |  | 0.050 |  |  |  |  |  |  | 0.139 |
|  | IMD 1 | 64.0 | [59.5, 68.9] | 81.1 | [75.5, 87.1] | 85.1 | [79.3, 91.2] |  | 74.6 | [70.4, 79.1] | 91.0 | [86.0, 96.2] | 99.3 | [94.1, 104.9] |  |
|  | IMD 2 | 104.6 | [97.8, 111.8] | 108.6 | [101.8, 116.0] | 102.7 | [96.1, 109.7] |  | 87.5 | [82.9, 92.5] | 106.0 | [100.5, 111.7] | 114.5 | [108.8, 120.6] |  |
|  | IMD 3 | 102.7 | [96.1, 109.7] | 126.3 | [118.5, 134.7] | 130.8 | [122.8, 139.3] |  | 98.9 | [93.8, 104.2] | 115.6 | [109.9, 121.7] | 123.9 | [117.9, 130.2] |  |
|  | IMD 4 | 112.7 | [105.5, 120.3] | 131.5 | [123.4, 140.1] | 141.7 | [133.2, 150.9] |  | 100.3 | [95.2, 105.7] | 118.5 | [112.7, 124.7] | 128.9 | [122.7, 135.3] |  |
|  | IMD 5 | 111.6 | [104.6, 119.2] | 141.1 | [132.4, 150.3] | 146.8 | [138.0, 156.2] |  | 101.0 | [95.8, 106.4] | 117.9 | [112.1, 124.0] | 134.0 | [127.6, 140.7] |  |
| Model 6 |  |  |  |  |  |  |  | 0.050 |  |  |  |  |  |  | 0.153 |
|  | IMD 1 | 86.5 | [79.9, 93.7] | 110.2 | [102.0, 119.0] | 116.0 | [107.5, 125.2] |  | 80.1 | [75.6, 84.9] | 97.8 | [92.5, 103.5] | 107.0 | [101.3, 113.0] |  |
|  | IMD 2 | 121.8 | [113.0, 131.3] | 142.3 | [132.2, 153.2] | 148.3 | [137.9, 159.4] |  | 94.0 | [89.0, 99.4] | 114.0 | [108.1, 120.2] | 123.4 | [117.2, 129.9] |  |
|  | IMD 3 | 139.7 | [129.8, 150.3] | 172.4 | [160.6, 185.2] | 178.8 | [166.7, 191.8] |  | 106.3 | [100.9, 112.2] | 124.5 | [118.3, 131.1] | 133.6 | [127.1, 140.4] |  |
|  | IMD 4 | 153.7 | [142.9, 165.2] | 179.6 | [167.3, 192.7] | 193.9 | [180.9, 207,9] |  | 108.0 | [102.5, 113.8] | 127.7 | [121.4, 134.4] | 138.9 | [132.2, 146.0] |  |
|  | IMD 5 | 152.5 | [141.8, 163.9] | 192.8 | [179.7, 206.8] | 200.8 | [187.4, 215.3] |  | 108.8 | [103.2, 114.7] | 127.0 | [120.7, 133.6] | 144.7 | [137.5, 151.7] |  |
| **Within patients ≥80y old** | |  |  |  |  |  |  |  |  |  |  |  |  |  |  |
| Model 5 |  |  |  |  |  |  |  | 0.590 |  |  |  |  |  |  | 0.260 |
|  | IMD 1 | 36.5 | [33.0, 40.3] | 53.4 | [48.8, 58.4] | 57.3 | [52.5, 62.6] |  | 36.9 | [34.1, 40.0] | 45.9 | [42.5, 49.5 | 48.9 | [45.4, 52.7] |  |
|  | IMD 2 | 47.9 | [43.8, 52.4] | 66.5 | [61.2, 72.2] | 73.3 | [67.6, 79.5] |  | 44.7 | [41.5, 48.1] | 52.0 | [48.5, 55.7] | 60.0 | [56.2, 64.2] |  |
|  | IMD 3 | 60.1 | [55.3, 65.4] | 81.5 | [75.4, 88.1] | 90.4 | [83.7, 97.5] |  | 50.9 | [47.5, 54.5] | 60.1 | [56.3, 64.1] | 65.1 | [61.1, 69.3] |  |
|  | IMD 4 | 67.0 | [61.7, 72.7] | 90.1 | [83.4, 97.2] | 102.5 | [95.1, 110.4] |  | 55.2 | [51.6, 59.1] | 63.3 | [59.4, 67.5] | 73.0 | [68.7, 77.6] |  |
|  | IMD 5 | 74.9 | [69.0, 81.2] | 93.1 | [86.3, 100.5] | 108.9 | [101.2, 117.3] |  | 57.1 | [53.3, 61.1] | 69.1 | [64.8, 73.5] | 78.9 | [74.3, 83.7] |  |
| Model 6 |  |  |  |  |  |  |  | 0.589 |  |  |  |  |  |  | 0.257 |
|  | IMD 1 | 41.5 | [37.5, 45.9] | 61.0 | [55.6, 66.9] | 65.9 | [60.2, 72.1] |  | 36.2 | [33.4, 39.2] | 44.9 | [41.6, 48.4] | 47.8 | [44.4, 51.5] |  |
|  | IMD 2 | 54.6 | [49.8, 59.9] | 76.1 | [69.9, 82.9] | 84.3 | [77.7, 91.6] |  | 43.7 | [40.6, 47.1] | 50.9 | [47.4, 54.5] | 58.7 | [54.9, 62.7] |  |
|  | IMD 3 | 68.9 | [63.1, 74.9] | 93.6 | [86.4, 101.5] | 104.4 | [96.5, 112.9] |  | 49.8 | [46.4, 53.4] | 58.7 | [55.0, 62.7] | 63.6 | [59.7, 67.7] |  |
|  | IMD 4 | 76.7 | [70.5, 83.3] | 103.7 | [95.8, 112.2] | 118.5 | [109.8, 127.9] |  | 54.0 | [50.5, 57.9] | 61.9 | [58.0, 66.0] | 71.3 | [67.1, 75.8] |  |
|  | IMD 5 | 86.1 | [79.2, 93.5] | 107.5 | [99.4, 116.3] | 126.4 | [117.1, 136.4] |  | 55.8 | [52.1, 59.8] | 67.4 | [63.3, 71.8] | 77.0 | [72.5, 81.7] |  |
| **Within females** | |  |  |  |  |  |  |  |  |  |  |  |  |  |  |
| Model 7 |  |  |  |  |  |  |  | 0.001 |  |  |  |  |  |  | <0.001 |
|  | IMD 1 | 23.5 | [22.5, 24.5] | 29.2 | [28.1, 30.4] | 31.1 | [30.0, 32.4] |  | 27.9 | [26.8, 28.9] | 36.6 | [35.4, 37.9] | 39.8 | [38.5, 41.1] |  |
|  | IMD 2 | 30.3 | [29.1, 31.5] | 36.0 | [34.7, 37.3] | 37.8 | [36.5, 39.2] |  | 31.1 | [30.0, 32.2] | 38.8 | [37.6, 40.1] | 42.8 | [41.5, 44.1] |  |
|  | IMD 3 | 35.0 | [33.7, 36.3] | 42.5 | [41.1, 44.0] | 45.9 | [44.4, 47.5] |  | 33.2 | [32.1, 34.3] | 40.6 | [39.3, 41.8] | 44.9 | [43.6, 46.3] |  |
|  | IMD 4 | 37.4 | [36.1, 38.7] | 45.0 | [43.5, 46.5] | 50.3 | [48.7, 51.9] |  | 33.4 | [32.4, 34.5] | 40.1 | [38.9, 41.3] | 46.5 | [45.2, 47.8] |  |
|  | IMD 5 | 38.6 | [37.2, 39.9] | 45.7 | [44.2, 47.2] | 53.1 | [51.4, 54.8] |  | 32.2 | [31.2, 33.3] | 39.4 | [38.2, 40.6] | 47.1 | [45.8, 48.4] |  |
| Model 8 |  |  |  |  |  |  |  | <0.001 |  |  |  |  |  |  | <0.001 |
|  | IMD 1 | 18.1 | [17.2, 19.0] | 23.1 | [22.1, 24.2] | 25.1 | [24.0, 26.3] |  | 21.8 | [21.0, 22.8] | 29.3 | [28.3, 30.5] | 32.3 | [31.2, 33.5] |  |
|  | IMD 2 | 23.6 | [22.5, 24.7] | 28.3 | [27.1, 29.6] | 29.8 | [28.5, 31.1] |  | 24.7 | [23.7, 25.6] | 30.9 | [29.8, 32.0] | 34.1 | [32.9, 35.3] |  |
|  | IMD 3 | 27.6 | [26.4, 28.8] | 33.2 | [31.9, 34.6] | 35.5 | [34.1, 37.0] |  | 26.5 | [25.6, 27.5] | 32.1 | [31.0, 33.3] | 35.2 | [34.0, 36.4] |  |
|  | IMD 4 | 29.8 | [28.6, 31.1] | 35.2 | [33.8, 36.6] | 38.6 | [37.1, 40.1] |  | 26.9 | [26.0, 27.9] | 31.7 | [30.6, 32.8] | 36.1 | [34.9, 37.3] |  |
|  | IMD 5 | 31.1 | [29.8, 32.4] | 35.9 | [34.5, 37.0] | 40.7 | [39.2, 42.4] |  | 26.2 | [25.3, 27.2] | 31.2 | [30.2, 32.3] | 36.5 | [35.3, 37.7] |  |
| **Within males** | |  |  |  |  |  |  |  |  |  |  |  |  |  |  |
| Model 7 |  |  |  |  |  |  |  | <0.001 |  |  |  |  |  |  | <0.001 |
|  | IMD 1 | 23.7 | [22.4, 25.1] | 28.9 | [27.4, 30.4] | 29.4 | [27.9, 30.9] |  | 22.1 | [21.2, 23.0] | 26.5 | [25.5, 27.5] | 28.1 | [27.1, 29.1] |  |
|  | IMD 2 | 31.5 | [29.9, 33.1] | 35.9 | [34.2, 37.6] | 38.8 | [37.0, 40.6] |  | 25.0 | [24.1, 26.0] | 29.6 | [28.6, 30.7] | 33.3 | [32.2, 34.4] |  |
|  | IMD 3 | 37.6 | [35.8, 39.4] | 44.2 | [42.3, 46.3] | 48.9 | [46.8, 51.1] |  | 27.0 | [26.0, 28.0] | 33.3 | [32.2, 34.4] | 36.7 | [35.5, 37.8] |  |
|  | IMD 4 | 39.6 | [37.8, 41.5] | 47.3 | [45.3, 49.4] | 52.9 | [50.7, 55.2] |  | 27.6 | [26.6, 28.6] | 34.2 | [33.1, 35.3] | 40.1 | [38.9, 41.4] |  |
|  | IMD 5 | 39.8 | [38.0, 41.7] | 49.1 | [47.0, 51.2] | 54.7 | [52.5, 57.1] |  | 26.8 | [25.9, 27.7] | 33.1 | [32.0, 34.2] | 40.7 | [39.5, 41.9] |  |
| Model 8 |  |  |  |  |  |  |  | 0.031 |  |  |  |  |  |  | <0.001 |
|  | IMD 1 | 18.1 | [17.0, 19.2] | 22.3 | [21.0, 23.6] | 22.8 | [21.6, 24.1] |  | 15.6 | [14.9, 16.3] | 18.9 | [18.1, 19.6] | 20.1 | [19.4, 20.9] |  |
|  | IMD 2 | 24.1 | [22.8, 25.4] | 27.3 | [25.9, 28.8] | 29.4 | [27.9, 30.9] |  | 17.7 | [17.0, 18.5] | 20.8 | [20.1, 21.7] | 23.3 | [22.5, 24.2] |  |
|  | IMD 3 | 28.7 | [27.3, 30.2] | 33.2 | [31.6, 34.9] | 36.2 | [34.5, 38.0] |  | 19.1 | [18.4, 19.9] | 23.1 | [22.3, 24.0] | 25.0 | [24.2, 25.9] |  |
|  | IMD 4 | 30.4 | [28.9, 31.9] | 35.4 | [33.7, 37.2] | 38.8 | [37.0, 40.7] |  | 19.7 | [18.9, 20.4] | 23.7 | [22.8, 24.5] | 27.1 | [26.2, 28.1] |  |
|  | IMD 5 | 30.6 | [29.1, 32.2] | 36.8 | [35.0, 38.6] | 40.0 | [38.2, 42.0] |  | 19.3 | [18.5, 20.0] | 22.9 | [22.1, 23.7] | 27.5 | [26.6, 28.4] |  |

Rates obtained from multilevel negative binomial regression models

IMD: Index of Multiple Deprivation; IMD 1: Most deprived Layer Super Output Area, IMD 5: Least deprived Layer Super Output Area

* Likelihood ratio test testing the interaction terms between IMD and year of joint replacement.

Model 1: Unadjusted; Model 2: Adjusted for patient age; Model 3: Adjusted for patient sex; Model 4: Adjusted for patient age and sex

Model 5: Unadjusted; Model 6: Adjusted for patient sex

Model 7: Unadjusted; Model 8: Adjusted for patient age.

**Table B.** Adjusted rates of joint replacement by patient sex in the overall population in 2007, 2012 and 2017

|  |  | **Hip** |  |  |  |  |  |  | **Knee** |  |  |  |  |  |  |
| --- | --- | --- | --- | --- | --- | --- | --- | --- | --- | --- | --- | --- | --- | --- | --- |
|  |  | **2007** |  | **2012** |  | **2017** |  |  | **2007** |  | **2012** |  | **2017** |  |  |
|  |  | **Rate** | **95% CI** | **Rate** | **95% CI** | **Rate** | **95% CI** | **p-value*** | **Rate** | **95% CI** | **Rate** | **95% CI** | **Rate** | **95% CI** | **p-value*** |
| **Overall** | |  |  |  |  |  |  |  |  |  |  |  |  |  |  |
| Model 1 |  |  |  |  |  |  |  | 0.151 |  |  |  |  |  |  | <0.001 |
|  | Female | 38.0 | [37.1, 38,8] | 45.7 | [44.7, 46.8] | 50.5 | [49.4, 51.7] |  | 26.1 | [25.6, 26.6] | 31.9 | [31.3, 32.5] | 36.5 | [35.9, 37.2] |  |
|  | Male | 28.8 | [28.1, 29.5] | 34.4 | [33.6, 35.3] | 37.8 | [36.9, 38.7] |  | 29.8 | [29.3, 30.4] | 36.9 | [36.2, 37.5] | 41.9 | [41.2, 42.6] |  |
| Model 2 |  |  |  |  |  |  |  | 0.174 |  |  |  |  |  |  | <0.001 |
|  | Female | 43.8 | [42.8, 44.9] | 52.8 | [51.5, 54.0] | 58.3 | [56.9, 59.7] |  | 27.2 | [26.7, 27.8] | 33.3 | [32.6, 33.9] | 38.2 | [37.4, 38.9] |  |
|  | Male | 33.2 | [32.4, 34.1] | 39.7 | [38.7, 40.7] | 43.6 | [42.5, 44.7] |  | 31.2 | [30.6, 31.8] | 38.5 | [37.8, 39.3] | 43.7 | [42.9, 44.6] |  |
| Model 3 |  |  |  |  |  |  |  | <0.001 |  |  |  |  |  |  | <0.001 |
|  | Female | 29.4 | [28.6, 30.3] | 35.2 | [34.3, 36.2] | 38.5 | [37.5, 39.6] |  | 19.9 | [19.5, 20.4] | 23.9 | [23.4, 24.4] | 27.0 | [26.5, 27.6] |  |
|  | Male | 23.1 | [22.3, 23.7] | 27.1 | [26.3, 27.9] | 29.2 | [28.4, 30.0] |  | 22.2 | [21.7, 22.7] | 27.2 | [26.6, 27.7] | 30.5 | [29.9, 31.2] |  |
| Model 4 |  |  |  |  |  |  |  | 0.001 |  |  |  |  |  |  | <0.001 |
|  | Female | 33.9 | [32.9, 34.9] | 40.5 | [39.3, 41.7] | 44.2 | [43.0, 45.5] |  | 20.8 | [20.3, 21.3] | 24.9 | [24.4, 25.5] | 28.2 | [27.6, 28.8] |  |
|  | Male | 26.5 | [25.7, 27.3] | 31.1 | [30.2, 32.0] | 33.5 | [32.6, 34.5] |  | 23.2 | [22.6, 23.7] | 28.3 | [27.7, 29.0] | 31.9 | [31.2, 32.6] |  |

Rates obtained from multilevel negative binomial regression models

* Likelihood ratio test testing the interaction terms between patient sex and year of joint replacement.

Model 1: Unadjusted; Model 2: Adjusted for Index of Multiple Deprivation; Model 3: Adjusted for patient age; Model 4: Adjusted for Index of Multiple Deprivation and patient age

**Table C.** Adjusted rates of joint replacement by patient age in the overall population in 2007, 2012 and 2017

|  |  | **Hip** |  |  |  |  |  |  | **Knee** |  |  |  |  |  |  |
| --- | --- | --- | --- | --- | --- | --- | --- | --- | --- | --- | --- | --- | --- | --- | --- |
|  |  | **2007** |  | **2012** |  | **2017** |  |  | **2007** |  | **2012** |  | **2017** |  |  |
|  |  | **Rate** | **95% CI** | **Rate** | **95% CI** | **Rate** | **95% CI** | **p-value** | **Rate** | **95% CI** | **Rate** | **95% CI** | **Rate** | **95% CI** | **p-value** |
| **Overall** | |  |  |  |  |  |  |  |  |  |  |  |  |  |  |
| Model 1 |  |  |  |  |  |  |  | <0.001 |  |  |  |  |  |  | <0.001 |
|  | Within patients <60y old | 20.0 | [19.3, 20.7] | 22.7 | [22.0, 23.5] | 25.4 | [24.6, 26.2] |  | 45.0 | [44.0, 46.1] | 55.0 | [53.7, 56.2] | 64.0 | [62.6, 65.4] |  |
|  | Within patients 60-70y old | 51.1 | [49.6, 52.7] | 57.9 | [56.3, 59.6] | 64.4 | [62.6, 66.3] |  | 72.6 | [70.9, 74.3] | 86.3 | [84.4, 88.3] | 94.8 | [92.7, 96.9] |  |
|  | Within patients 70-80y old | 80.3 | [77.9, 82.7] | 97.8 | [95.1, 100.7] | 102.7 | [99.9, 105.7] |  | 40.6 | [39.4, 41.8] | 48.4 | [47.1, 49.8] | 54.9 | [53.5, 56.3] |  |
|  | Within patients ≥80y old | 47.2 | [[45.5, 48.9] | 63.6 | [61.6, 65.7] | 72.2 | [69.9, 74.4] |  | 14.2 | [13.8, 14.7] | 19.3 | [18.8, 19.9] | 22.7 | [22.1, 23.3] |  |
| Model 2 |  |  |  |  |  |  |  | <0.001 |  |  |  |  |  |  | <0.001 |
|  | Within patients <60y old | 14.1 | [13.6. 14.6] | 16.0 | [15.4, 16.5] | 17.9 | [17.3, 18.5] |  | 40.2 | [39.2, 41.2] | 49.0 | [47.8, 50.2] | 57.1 | [55.8, 58.4] |  |
|  | Within patients 60-70y old | 35.9 | [34.8, 37.1] | 40.7 | [39.5, 42.0] | 45.3 | [44.0, 46.7] |  | 64.8 | [63.2, 66.5] | 77.0 | [75.2, 78.9] | 84.4 | [82.5, 86.4] |  |
|  | Within patients 70-80y old | 56.6 | [54.9, 58.4] | 68.8 | [66.8, 71.0] | 72.1 | [70.0, 74.3] |  | 36.3 | [35.2, 37.4] | 43.2 | [42.0, 44.5] | 48.9 | [47.6, 50.3] |  |
|  | Within patients ≥80y old | 33.4 | [32.2, 34.6] | 44.8 | [43.3, 46.4] | 50.8 | [49.1, 52.4] |  | 12.7 | [12.3, 13.1] | 17.3 | [16.8, 17.8] | 20.3 | [19.7, 20.8] |  |
| Model 3 |  |  |  |  |  |  |  | <0.001 |  |  |  |  |  |  | <0.001 |
|  | Within patients <60y old | 23.8 | [22.9, 24.6] | 27.0 | [26.1, 27.9] | 30.2 | [29.2, 31.2] |  | 47.9 | [46.8, 49.1] | 58.5 | [57.2, 59.8] | 68.1 | [66.6, 69.7] |  |
|  | Within patients 60-70y old | 60.6 | [58.8, 62.5] | 68.7 | [66.7, 70.8] | 76.4 | [74.2, 78.7] |  | 77.1 | [75.2, 78.9] | 91.7 | [89.6, 93.8] | 100.7 | [98.5, 103.0] |  |
|  | Within patients 70-80y old | 94.6 | [91.7, 97.5] | 115.5 | [112.1, 119.0] | 121.4 | [117.9, 125.0] |  | 42,6 | [41.3, 43.9] | 50.9 | [49.5, 52.3] | 57.8 | [56.3, 59.3] |  |
|  | Within patients ≥80y old | 54.2 | [52.2, 56.2] | 73.4 | [71.0, 75.9] | 83.7 | [81.0, 86.4] |  | 15.2 | [14.7, 15.6] | 20.6 | [20.0, 21.2] | 24.2 | [23.6, 24.8] |  |
| Model 4 |  |  |  |  |  |  |  | <0.001 |  |  |  |  |  |  | <0.001 |
|  | Within patients <60y old | 16.7 | [16.1, 17.3] | 18.9 | [18.3, 19.6] | 21.2 | [20.5, 21.9] |  | 42.7 | [41.7, 43.8] | 52.1 | [50.9, 53.4] | 60.7 | [59.3, 62.2] |  |
|  | Within patients 60-70y old | 42.6 | [41.2, 44.0] | 48.2 | [46.7, 49.7] | 53.6 | [52.0, 55.3] |  | 68.7 | [67.1, 70.5] | 81.7 | [79.8, 83.7] | 89.7 | [87.6, 91.8] |  |
|  | Within patients 70-80y old | 66.6 | [64.5, 68.7] | 81.1 | [78.6, 83.7] | 85,1 | [82.5, 87.8] |  | 38.0 | [36.9, 39.2] | 45.4 | [44.1, 46.7] | 51.5 | [50.1, 52.9] |  |
|  | Within patients ≥80y old | 38.2 | [36.8. 39.7] | 51.6 | [49.9, 53.4] | 58.8 | [56.8, 60.8] |  | 13.5 | [13.1, 14.0] | 18.4 | [17.9, 18.9] | 21.6 | [21.0, 22.2] |  |

Rates obtained from multilevel negative binomial regression models

* Likelihood ratio test testing the interaction terms between patient age and year of joint replacement.

Model 1: Unadjusted; Model 2: Adjusted for Index of Multiple Deprivation; Model 3: Adjusted for patient sex; Model 4: Adjusted for Index of Multiple Deprivation and patient sex

**Figure A1.** Patient flow Diagram for hip replacement

**Figure A2.** Patient flow Diagram for knee replacement

**Figure B.** Rates and 95% Confidence Intervals of joint replacement provision by sex and year of procedure


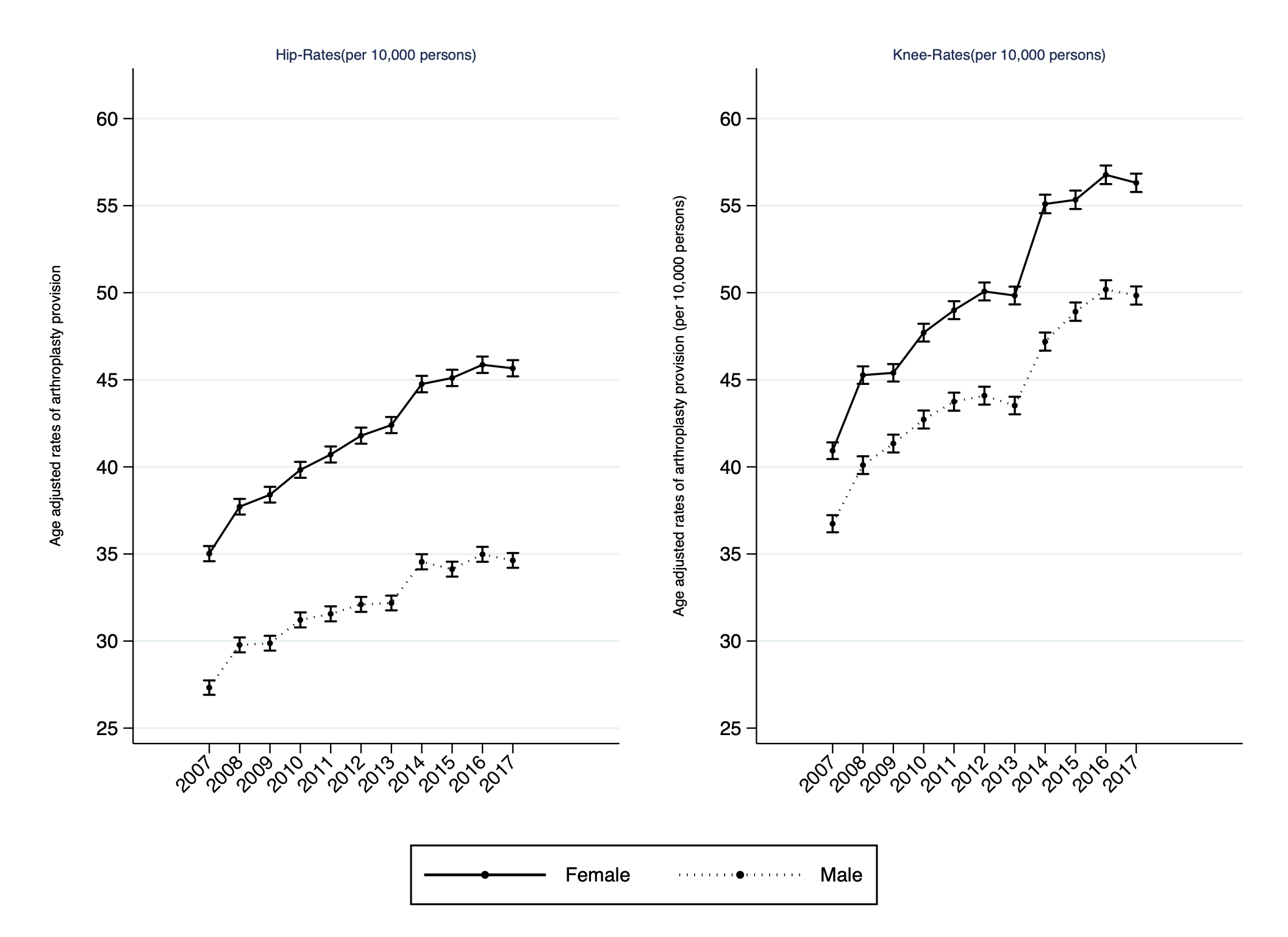


**Figure C.** Rates ratio and 95% Confidence Intervals of joint replacement for males (reference: females)-adjusted for age, deprivation,, and area of residence (Lower Layer Super Output Area)


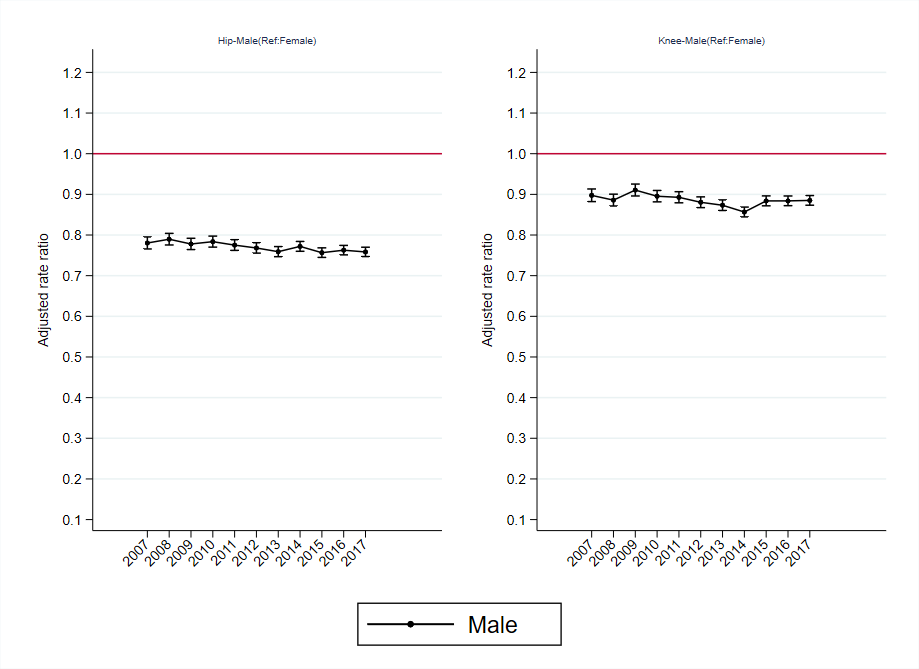


**Figure D.** Rates and 95% Confidence Intervals of joint replacement provision by age and year of procedure


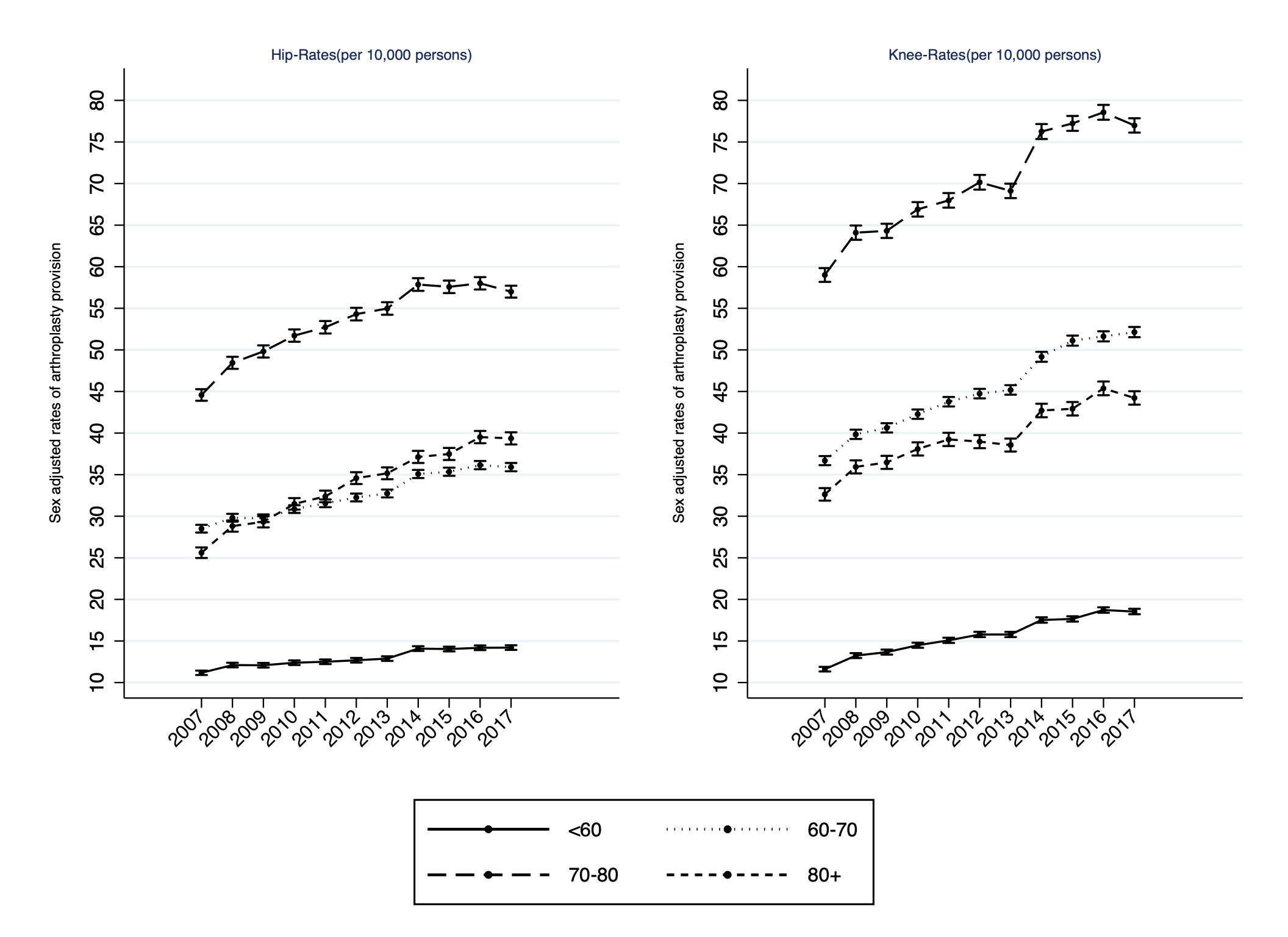


**Figure E.** Rates ratio and 95% Confidence Intervals of joint replacement between age groups (reference: < 60 years old) adjusted for sex, deprivation and area of residence (Lower Layer Super Output Area)


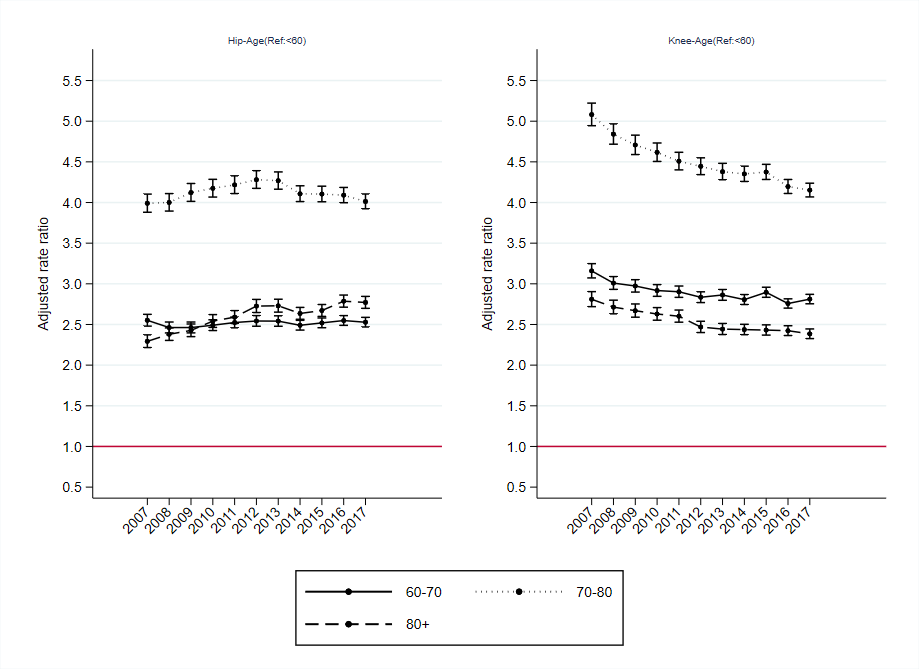


**Figure F**. Rates with 95% Confidence Intervals of joint replacement provision by area of residence deprivation level and year of procedure by sex


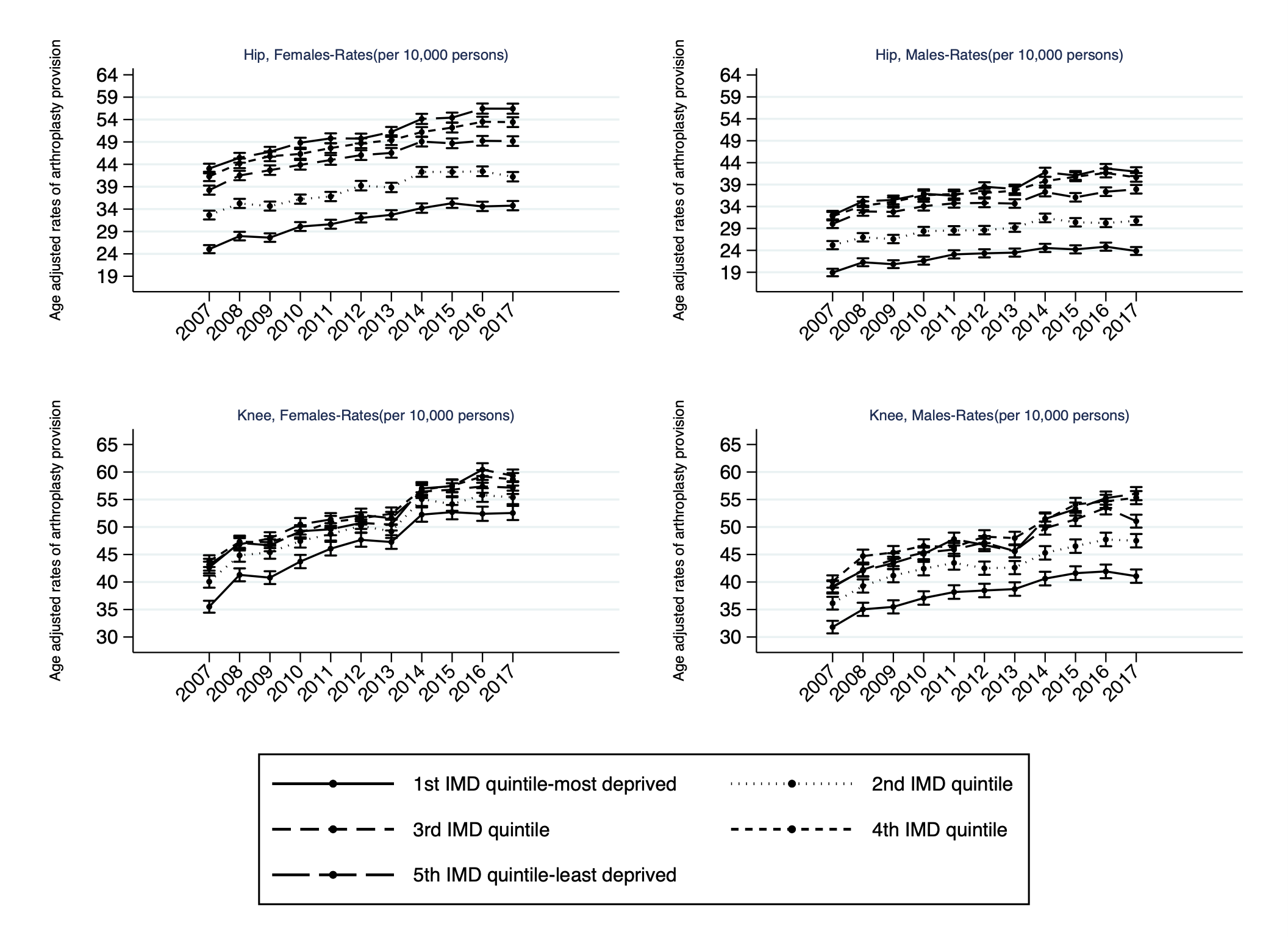


**Figure G.** Rates ratio and 95% Confidence Intervals of joint replacement between level of area of residence deprivation level (reference: IMD=5) stratified by sex and-adjusted for age, and area of residence (Lower Layer Super Output Area)


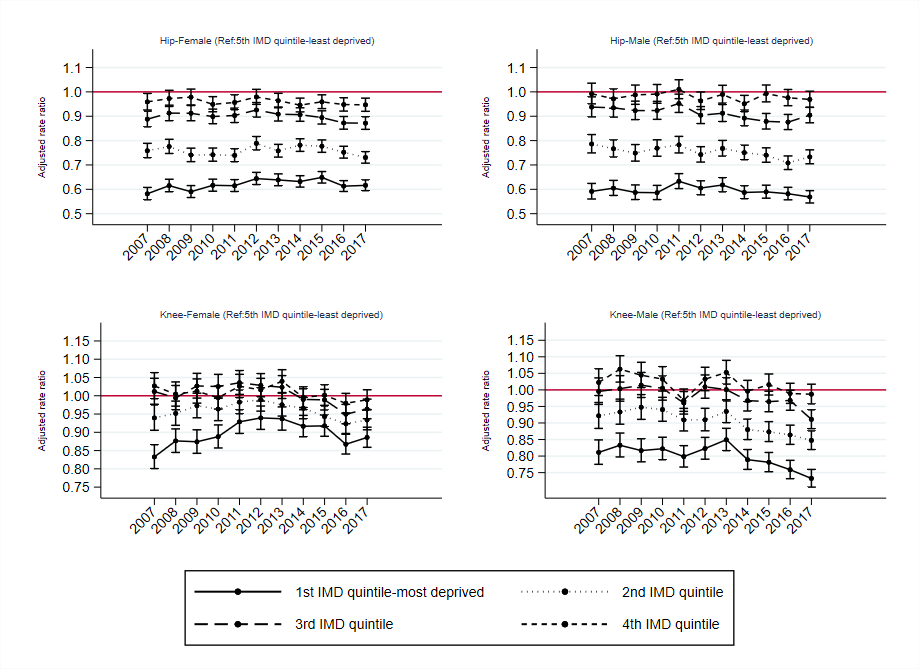


**Figure H.** Rates ratio and 95% Confidence Intervals of joint replacement between level of area of residence deprivation level (reference: IMD=5) stratified by type of healthcare provider and-adjusted for age, sex and area of residence (Lower Layer Super Output Area)
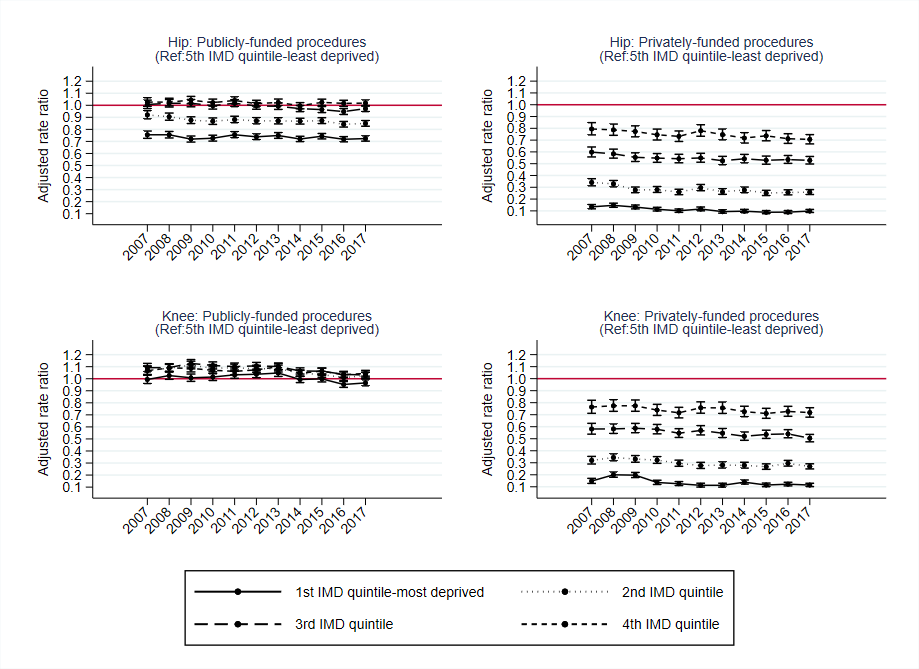


**Figure I1.** Rates with 95% Confidence Intervals of hip replacement provision by area of residence deprivation level and year of procedure stratified by age groups


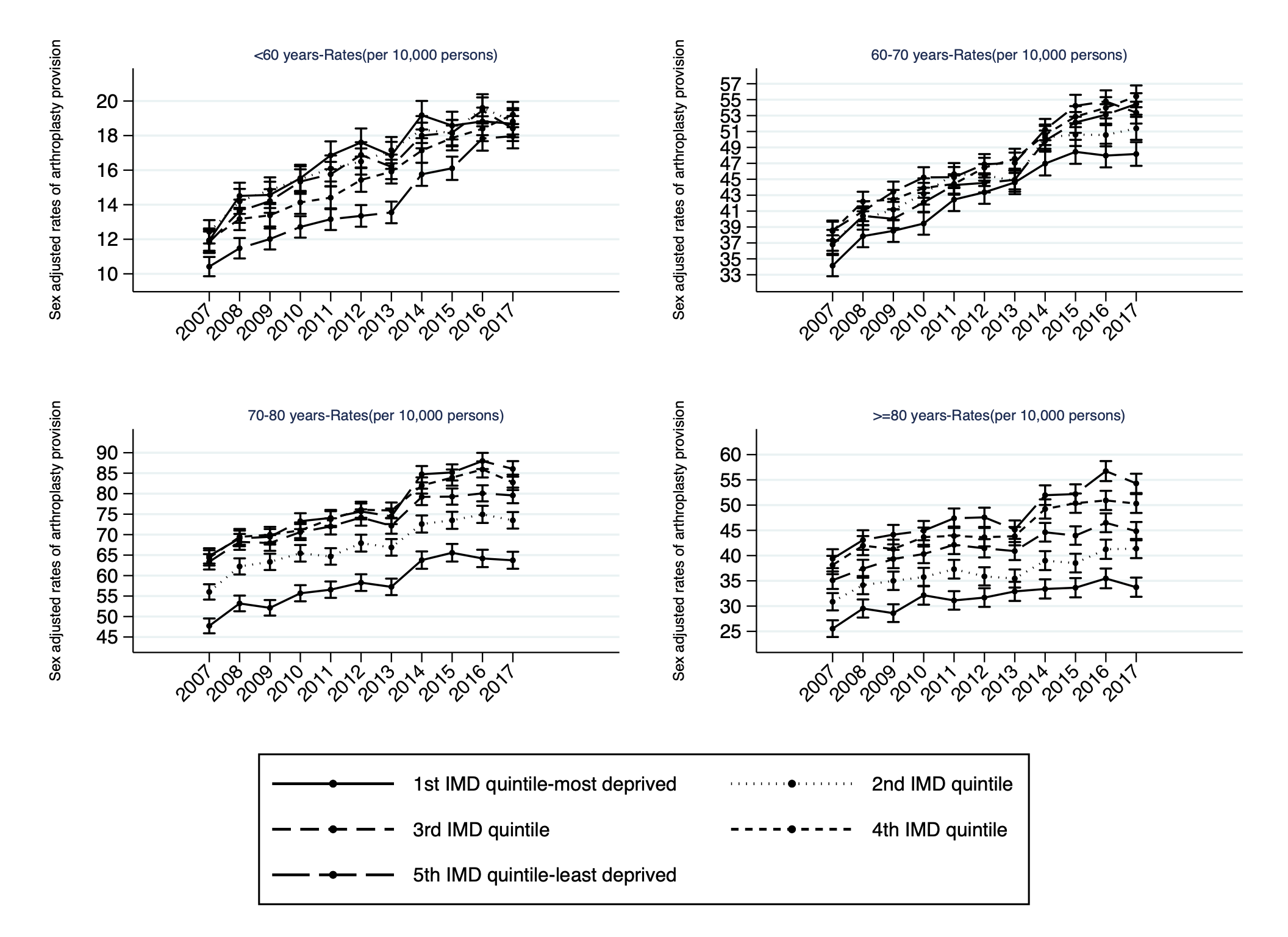


**Figure I2.** Rates with 95% Confidence Intervals of knee replacement provision by area of residence deprivation level and year of procedure stratified by age groups


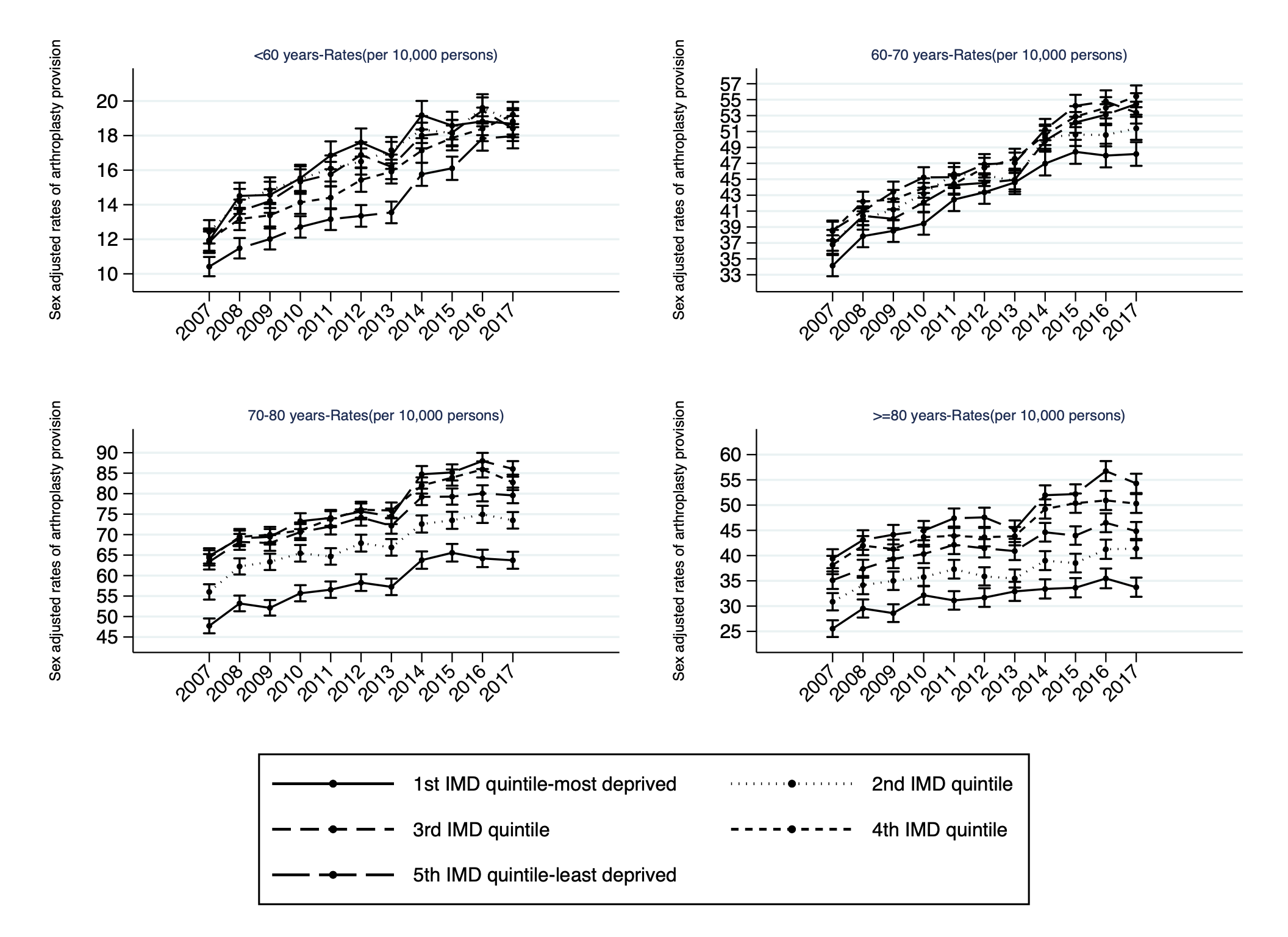


**Figure J1.** Rates ratio and 95% Confidence Intervals of hip replacement between level of area of residence deprivation level (reference: IMD=5) stratified by age groups and-adjusted for sex, and area of residence (Lower Layer Super Output Area)


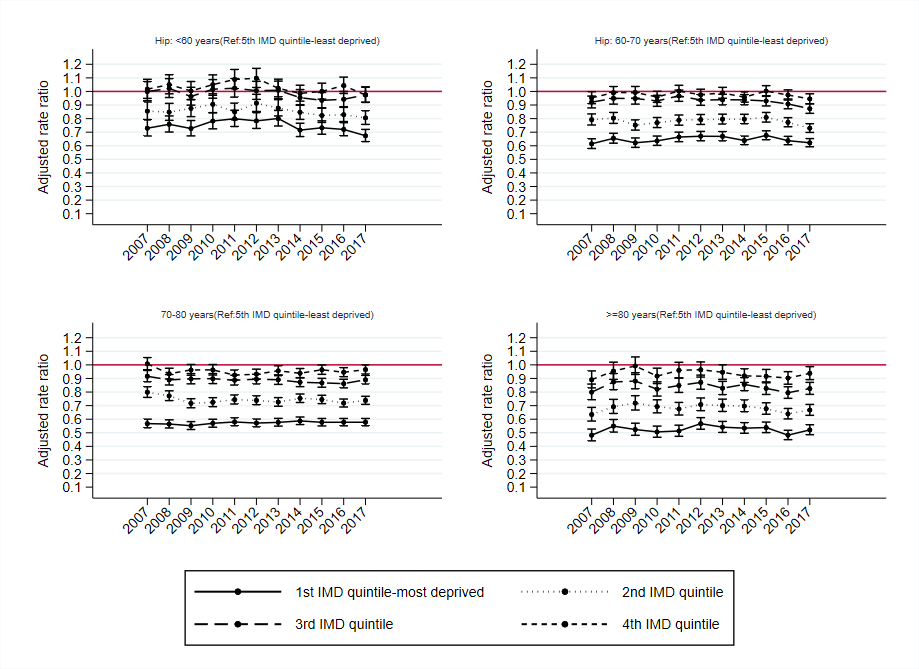


**Figure J2.** Rates ratioand 95% Confidence Intervals of knee replacement between level of area of residence deprivation level (reference: IMD=5) stratified by age groups and-adjusted for sex, and area of residence (Lower Layer Super Output Area)


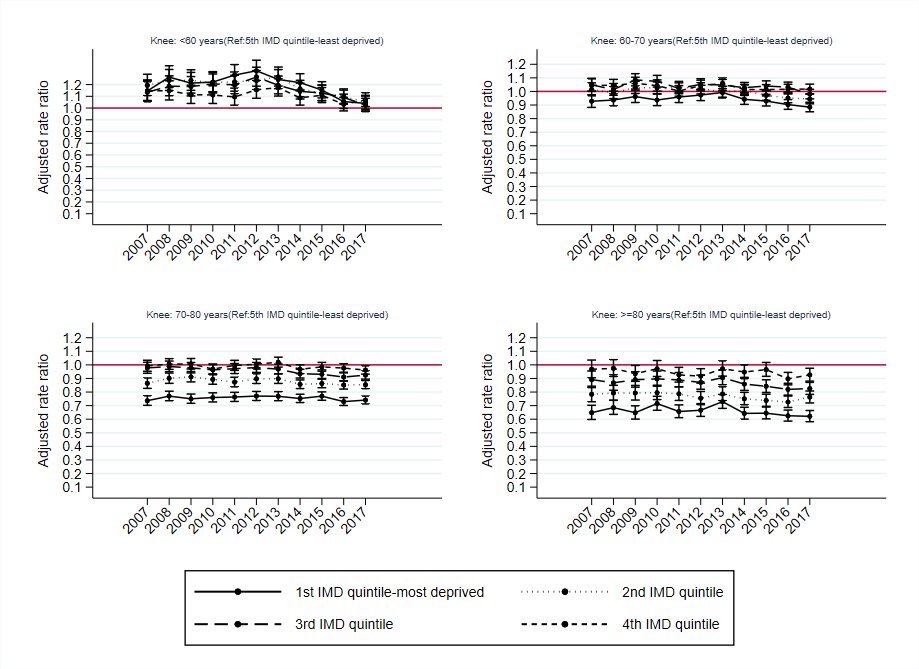

Supplement: S1 Appendix — Figure A1. Patient flow diagram for hip replacement. Figure A2. Patient flow diagram for knee replacement. Figure B. Rates and 95% confidence intervals of joint replacement provision by sex and year of procedure. Figure C. Rates ratio and 95% confidence intervals of joint replacement for males (reference: females) adjusted for age, deprivation, and area of residence (Lower Layer Super Output Area). Figure D. Rates and 95% confidence intervals of joint replacement provision by age and year of procedure. Figure E. Rates ratio and 95% confidence intervals of joint replacement between age groups (reference: <60 years old) adjusted for sex, deprivation, and area of residence (Lower Layer Super Output Area). Figure F. Rates with 95% confidence intervals of joint replacement provision by area of residence deprivation level and year of procedure by sex. Figure G. Rates ratio and 95% confidence intervals of joint replacement between level of area of residence deprivation level (reference: IMD = 5) stratified by sex and adjusted for age and area of residence (Lower Layer Super Output Area). Figure H. Rates ratio and 95% confidence intervals of joint replacement between level of area of residence deprivation level (reference: IMD = 5) stratified by type of healthcare provider and adjusted for age, sex, and area of residence (Lower Layer Super Output Area). Figure I1. Rates with 95% confidence intervals of hip replacement provision by area of residence deprivation level and year of procedure stratified by age groups. Figure I2. Rates with 95% confidence intervals of knee replacement provision by area of residence deprivation level and year of procedure stratified by age groups. Figure J1. Rates ratio and 95% confidence intervals of hip replacement between level of area of residence deprivation level (reference: IMD = 5) stratified by age groups and adjusted for sex and area of residence (Lower Layer Super Output Area). Figure J2. Rates ratio and 95% confidence intervals of knee [file pmed.1004210.s002.docx]
